# Plasma dephosphorylated-uncarboxylated Matrix Gla-Protein (dp-ucMGP): reference intervals in Caucasian adults and diabetic kidney disease biomarker potential

**DOI:** 10.1038/s41598-019-54762-2

**Published:** 2019-12-05

**Authors:** Tomás Patrick Griffin, Md Nahidul Islam, Deirdre Wall, John Ferguson, Damian Gerard Griffin, Matthew Dallas Griffin, Paula M. O’Shea

**Affiliations:** 1 Centre for Endocrinology, Diabetes and Metabolism, Saolta University Health Care Group, Galway University Hospitals, Galway, Ireland; 2 Regenerative Medicine Institute (REMEDI) at CÚRAM SFI Research Centre, School of Medicine, National University of Ireland Galway, Galway, Ireland; 3 Department of Clinical Biochemistry, Saolta University Health Care Group, Galway University Hospitals, Galway, Ireland; 4 School of Mathematics, Statistics and Applied Mathematics, National University of Ireland Galway, Galway, Ireland; 5 Health Research Board (HRB), Clinical Research Facility, National University of Ireland Galway, Galway, Ireland; 6 Department of Nephrology, Saolta University Health Care Group, Galway University Hospitals, Galway, Ireland

**Keywords:** Predictive markers, Chronic kidney disease, Biochemistry, Predictive markers, Diabetes complications

## Abstract

Recent studies suggest a possible association between dephosphorylated-uncarboxylated MGP (dp-ucMGP) and glomerular filtration rate (GFR). This study aimed to establish normative data in an adult Caucasian population and to explore the potential utility of dp-ucMGP in patients with diabetes mellitus (DM) with and without diabetic kidney disease (DKD). Healthy volunteers (HVs) (cross-sectional study) and participants with DM (prospective cohort study) were recruited. Plasma dp-ucMGP was measured using the IDS®-iSYS Ina Ktif (dp-ucMGP) assay. Of the HVs recruited (n = 208), 67(32.2%) were excluded leaving a reference population of 141(67.8%) metabolically healthy participants with normal kidney function. Plasma dp-ucMGP RIs were <300–532 pmol/L. There were 100 eligible participants with DKD and 92 with DM without DKD. For the identification of participants with DKD, the area under the receiver operating characteristic curve (AUC) for dp-ucMGP was 0.842 (95%CI:0.799–0.880; p < 0.001). Plasma dp-ucMGP demonstrated similar ability to urine albumin:creatinine ratio (uACR) to detect participants with DM and renal function decline. Among patients with DM, there was a negative correlation between natural log (LN) dp-ucMGP and eGFR (r = −0.7041; p < 0.001) and rate of change in renal function [%change (r = −0.4509; p < 0.001)] and a positive correlation between LN dp-ucMGP and LN uACR (r = 0.3392; p < 0.001). These results suggest the potential for plasma dp-ucMGP with well-defined RIs to identify adults at high risk for vascular disease in the context of progressive DKD.

## Introduction

Matrix Gla-protein (MGP), a 10-kDA secreted protein^1^, is a member of the family of vitamin K-dependent proteins (Gla [γ-carboxyglutamate] proteins)^2^. It is expressed by arterial medial vascular smooth muscle cells, fibroblasts, chondrocytes and endothelial cells^3^ in a variety of tissues including the arterial wall, heart, lungs and kidneys^4^. MGP is a potent inhibitor of vascular calcification^5^. Mice that lack MGP develop to term but die within two months due to arterial calcification which leads to blood-vessel rupture^6^. Keutel syndrome, a rare autosomal recessive disorder, is caused by a loss-of-function mutation of MGP that results in abnormal calcification^7^. Increased vascular calcification leads to increased MGP transcription and production of dephosphorylated-uncarboxylated MGP (dp-ucMGP)^8^. Post-translational modification of dp-ucMGP is necessary for it to acquire its full calcification inhibitory activity: dp-ucMGP (inactive) undergoes γ-glutamate carboxylation to form dephosphorylated-carboxylated MGP (dp-cMGP) (intermediate) followed by serine phosphorylation to form active phosphorylated-carboxylated MGP (p-cMGP). Vitamin K hydroquinone is an essential cofactor for the enzyme γ-glutamylcarboxylase that catalyses the conversion of dp-ucMGP to dp-cMGP. Plasma concentration of dp-ucMGP is considered to be a better indicator of vascular vitamin K status than other components of the MGP system and correlates with vascular stiffness in the general population and in patients with chronic kidney disease (CKD)^3,9,10^.

Patients with diabetes mellitus (DM) have increased risk of cardiovascular morbidity and mortality^11,12^. Cardiovascular disease (CVD) affects approximately 30% of all persons with type 2 DM (T2DM); reducing life expectancy by up to 10 years^12^. DM is the leading cause of CKD worldwide (known as diabetic kidney disease (DKD))^13^ and in conjunction with kidney disease is highly associated with vascular calcification^14^. In the population-based NHANES III study in those with T2DM, the subgroup with evidence of CKD accounted for most or all of the excess cardiovascular mortality risk compared to those without DM^15^. Elevated plasma dp-ucMGP was associated with vascular calcification in patients with different degrees of kidney disease^10,16^ and independently associated with lower renal function^9^. Plasma dp-ucMGP was also independently associated with peripheral vascular calcification^3^, carotid femoral pulse wave velocity^17^ and aortic pulse wave velocity^18^. Aortic pulse wave velocity is an independent predictor of cardiovascular morbidity and mortality^19^. Vitamin K supplementation (VKS) has been associated with a reduction in vascular calcification and plasma dp-ucMGP concentrations although further studies are needed^20^. Thus, measurement of plasma dp-ucMGP has the potential to be a marker of effective VKS. Finally, vitamin K-dependent proteins such as dp-ucMGP are associated with the combined endpoint of CVD or mortality^20^.

There is a significant unmet clinical need for the identification of biomarkers that serve as predictors of renal function decline^21^ and/or identification of patients at risk of CVD with differing degrees of renal impairment. To identify the utility of a biomarker in a disease process, it is important firstly to understand how this biomarker behaves in a healthy population with no underlying disease processes. To our knowledge, this is the first report to establish robust reference intervals (RIs) for dp-ucMGP measured using the IDS®-iSYS, an automated immunoassay analyzer based on chemiluminescent technology, in metabolically healthy adults with normal kidney function. Previous studies have established reference intervals for total MGP using a sandwich ELISA kit^22^ and explored the relationships between dp-ucMGP concentrations measured using a sandwich dual-antibody ELISA and coronary artery calcification and vitamin K status in healthy women^23,24^. The primary aims of this study were to establish normative data for dp-ucMGP in an Irish Caucasian population and to explore the promise of dp-ucMGP as a new biomarker in patients with DKD. The secondary aim was to compare plasma dp-ucMGP and urine albumin:creatinine ratio (uACR) in the identification of individuals with rapid decline in renal function.

## Methods

Ethical approval for this study was granted by the research ethics committees at Galway University Hospitals (GUH) and the National University of Ireland Galway (NUIG) (Ref GUH: C.A. 1404; Ref NUIG: 16-July-05). The ethical principles of this study are based on the recommendations set out in the Declaration of Helsinki. Informed written consent was obtained from all participants.

### Study design

A cross-sectional study was carried out between March 2016 and March 2018 at GUH/NUIG to recruit healthy volunteers (HVs). HVs were identified using posters displayed at GUH/NUIG. A prospective cohort study was designed to recruit participants with DM with and without DKD. Participants were recruited by convenience consecutive sampling at routine DM, nephrology and diabetic renal clinics^25^ at GUH.

### Reference population

Our reference population of metabolically healthy adults with normal kidney function has been previously described^26^. In brief, the stringent inclusion criteria for establishing reference intervals in our population (Table 1) included: signed informed consent and Caucasian ethnicity. The exclusion criteria included: on prescribed or over-the-counter medications (not including contraceptives) in the week preceding recruitment, previous or new diagnosis of DM or prediabetes, known diagnosis of cardiac, thyroid, liver or kidney disease, anaemia or unwell in the previous 2-weeks, non-Caucasian, clinical or laboratory parameters outside defined ranges (Table 1) or insufficient sample. In addition to the previously described exclusion criteria, any participant suspected of having metabolic bone disease at the time of sampling (low 25-hydroxyvitamin D(25(OH)D) <25 ng/mL and/or high intact parathyroid hormone (iPTH) concentration ≥65 ng/L) was also excluded. The number of participants meeting these criteria and included in the reference population was 141.

**Table 1:** Baseline characteristics and inclusion criteria for reference population (n = 141): correlation of characteristics with LN dp-ucMGP.

Parameter	Inclusion Criteria	Median (Range)	Correlation Coefficient*	P-Value
Age (years)	≥18	30.0 (18.1–62.2)	0.109	0.198
BMI (kg/m^2^)	≤32.5	24.0 (16.7–32.4)	0.305	**<0.001**
Pulse (beats per min)	N/A	68 (22–108)	0.123	0.145
SBP (mmHg)	<146	123 (93–145)	0.143	0.091
DBP (mmHg)	<89	75 (49–88)	0.165	0.051
HbA_1c_ (mmol/mol)	20–42^≠^	32 (21–39)	−0.014	0.876
CRP (mg/L)^	<10	0.7 (0.5–8.9)	0.33	**<0.001**
Sodium (mmol/L)	134–146^≠^	141 (134–145)	−0.041	0.63
Potassium (mmol/L)	3.5–5.2^≠^	4.3 (3.6–5.0)	−0.062	0.463
Chloride (mmol/L)	N/A	101 (94–105)	0.034	0.686
Urea (mmol/L)	N/A	5.0 (2.5–9.3)	−0.111	0.191
Creatinine (µmol/L)	45–110^≠^	76 (47–110)	−0.038	0.651
eGFR (ml/min/1.73 m^2^)	≥60	99 (65–133)	−0.198	**0.019**
Adj. Calcium (mmol/L)	2.15–2.51^≠^	2.31 (2.17–2.45)	0.078	0.361
Phosphate (mmol/L)	0.7–1.5	1.14 (0.71–1.46)	−0.064	0.454
Total Bilirubin (µmol/L)	≤23	8 (2–23)	−0.094	0.267
ALP (U/L)	<130	59 (29–111)	−0.001	0.986
ALT (U/L)	<1.5x URL (40) or <60	18 (7–48)	0.044	0.605
GGT (U/L)^	<3x URL (35) or <105	16 (6–83)	0.133	0.117
Cholesterol (mmol/L)	N/A	4.6 (2.9–7.0)	0.125	0.139
Triglycerides (mmol/L)^	N/A	0.9 (0.3–4.7)	0.056	0.509
HDL-C (mmol/L)	N/A	1.6 (0.7–3.1)	−0.004	0.959
LDL-C (mmol/L)	N/A	2.3 (1.1–4.2)	0.132	0.119
Free T4 (pmol/L)	10.5–24^≠^	16.1 (11.3–24.0)	−0.075	0.374
TSH (mIU/L)	0.27–4.78^≠^	1.92 (0.28–4.71)	0.159	0.06
iPTH (ng/L)^	<65	30.7 (8.1–62.3)	−0.07	0.406
25 (OH) D (ng/mL)^	≥25	52 (25–118)	−0.145	0.086
hsTnT (ng/L)^	<14	4 (4–10)	−0.089	0.297
NT-proBNP (ng/L)^	≤150	23.7 (9–150)	0.158	0.062
uACR (mg/mmol)^	N/A	0.63 (0.05–19.5)	0.196	**0.02**
WCC (10*^9^/L)	3–12^≠^	6.3 (3.1–11.6)	0.181	**0.033**
Haemoglobin (g/dL)	M > 13; F > 11	13.7 (11.3–16.8)	−0.044	0.608
Platelet Count (10*^9^/L)	128–450^≠^	248 (129–421)	0.172	**0.043**

BMI: body mass index; SBP: systolic blood pressure; DBP: diastolic blood pressure; HbA_1c_: glycated haemoglobin; CRP: C-reactive protein; eGFR: estimated glomerular filtration rate (CKD-EPI); Adj. Calcium: adjusted calcium, ALP: alkaline phosphatase; ALT: alanine aminotransferase; GGT: Gamma-glutamyl transferase; HDL-C: high-density lipoprotein cholesterol; LDL-C: low-density lipoprotein cholesterol; T4: thyroxine; TSH: thyroid stimulating hormone; iPTH: intact parathyroid hormone; 25(OH)D: 25 hydroxy vitamin D; hsTnT: high-sensitivity troponin T; NT-proBNP: n-terminal pro b-type natriuretic peptide; WCC: white cell count; uACR: urine albumin:creatinine ratio. ~Data is represented as median (range) to indicate the spread of results. *Pearson’s correlation. Significant p-values are highlighted in bold. ^Correlation between LN of the variable and LN dp-ucMGP. ^≠^Includes both the minimum and maximum value.

### Participants with DM and DKD

DKD was defined as a reduction in eGFR (<60 mL/min/1.73 m^2^), persistent albuminuria (uACR > 2.5 mg/mmol (males) or >3.5 mg/mmol (females) on ≥2 of the last 3 uACR measurements) and/or renal hyperfiltration >150 mL/min/1.73 m^2^. Participants with DM were divided into groups based on the presence or absence of DKD. The inclusion criteria were: known diagnosis of DM, age ≥18 years and signed informed consent. The exclusion criteria were: under active management for acute medical conditions (infection, cancer, acute cardiovascular event or haematological conditions) other than anaemia, haemoglobin <10 g/dL in the 3 months prior to study enrolment, Vitamin K antagonist therapy, CKD 5, renal transplantation or dialysis therapy.

### Data collection

All participants had baseline demographics and clinical characteristics recorded (Supplementary Methodology 1). Blood pressure (systolic [SBP], diastolic [DBP]) was measured in mm Hg using an automated oscillometric device (Omron®) in accordance with standard departmental operating procedures, after participants had been seated and at rest for five minutes. The reference population completed a detailed questionnaire to identify any known medical conditions, medication use and/or illness in the previous two weeks. For participants with DM, the type and duration of DM (years), past medical history and current medications were noted. Cardiovascular disease (CVD) is defined as a history of myocardial infarction, congestive cardiac failure or stroke. In addition, all serum creatinine values for the 10 years pre-enrolment and over the subsequent 2.5 years post-enrolment (or until death, dialysis or transplantation) were collated. For these serial serum creatinine values, eGFR was calculated using the 4-parameter CKD-EPI formula^27^.

### Laboratory sampling strategy

Blood (20 mL) was drawn from each participant and collected in appropriate specimen tubes (BD Vacutainer® blood collection tubes): potassium ethylenediaminetetraacetic acid (EDTA) tubes for measurement of plasma glycated haemoglobin (HbA_1c_), full blood count and plasma dp-ucMGP and plain (clotting) tubes for measurement of serum C-reactive protein (CRP), sodium, potassium, chloride, urea, creatinine, calcium, phosphate, liver function tests, cholesterol, thyroid function tests, iPTH, 25(OH)D, high sensitivity troponin t (hsTnT) and N-terminal pro b-type natriuretic peptide (NT-proBNP). A midstream urine sample was collected for measurement of uACR (Supplementary Methodology 2).

For measurement of plasma dp-ucMGP, whole blood collected in EDTA tubes was processed (centrifugation at 3000xg for 10 minutes at 4 °C and plasma separated from the cells within 1.5 h of blood draw), divided into aliquots and stored at −80 °C prior to batch analyses.

### Analytical methods

The IDS®-iSYS is an automated immunoassay analyzer based on chemiluminescent technology. The IDS®-iSYS Ina-Ktif (dp-ucMGP) is a sandwich assay employing two monoclonal murine antibodies, a magnetic particle, solid-phase capture dpMGP antibody and an acridinium-labelled tag ucMGP antibody. The quantity of light (expressed in relative light units) emitted by the acridinium label is directly proportional to the concentration of dp-ucMGP in the sample and measured by the system luminometer. All analyses were conducted in the Clinical Biochemistry Laboratory at GUH (accredited to ISO 15189:2012 standards for medical testing laboratories).

### Assessment of assay performance specifications

Linearity, precision, bias, the effect of haemolysis, sample stability and the effect of repeated freeze-thaw cycles on dp-ucMGP measurement were assessed (Supplementary Methodology 3).

### Statistical analyses

Microsoft® Excel 2016, GraphPad® Prism (Version 6.01) and R^28^ were used for data recording and statistical analyses.

#### Establishing reference intervals for dp-ucMGP

The frequency distribution for plasma dp-ucMGP was established. Reference values within the IDS-iSYS dp-ucMGP reportable range were used to establish the reference intervals. Plasma dp-ucMGP levels below the assay reportable range or lower limit of quantification (LLoQ) (300 pmol/L) were assigned the arbitrary figure of 299 pmol/L for statistical analyses. Normality was evaluated using the Anderson-Darling test. Outliers were assessed in accordance with the criteria of Dixon *et al*.^29^ and Reed *et al*.^30^ (Supplementary Methodology 4). As the data was not normally distributed, the International Federation of Clinical Chemistry and Laboratory Medicine (IFCC) recommended non-parametric method was used to establish the reference intervals. Plasma dp-ucMGP lower and upper reference limits were estimated at the 2.5^th^ and 97.5^th^ percentiles, respectively.

#### Diagnostic utility of dp-ucMGP in DKD

Continuous data were represented using means (standard deviations) where the data were normally distributed and median (min-max) for non-normally distributed data. For normally distributed data, comparison of means was made using ANOVA with Tukey’s post hoc multiple comparison test or Student’s T-test. For non-parametric data, Kruskal-Wallis multiple comparison test with Dunn’s post hoc multiple comparison test and Mann-Whitney U test were used. Categorical data were summarized with frequencies (percentages). Comparison of proportions were performed using the chi-squared test (with pairwise tests for independence for multiple comparisons). Pearson’s correlation was used to explore the relationships between continuous variables and clinical/laboratory parameters among HVs. P-values ≤0.05 were considered statistically significant.

Linear mixed-effects models (incorporating random within subject trajectories of eGFR over time) were used to generate individual specific eGFR slopes; both absolute change (mL/min/1.73 m^2^/year) and percentage change (% change/year). The slopes represented the change in renal function over time for each participant incorporating all eGFR measurements (before, after and at time of recruitment). Progressive renal function decline among participants with DM (decliners) was defined as either an absolute reduction in eGFR of ≥3.5 mL/min/1.73 m^2^/year^31^ or percentage eGFR loss of ≥3.3%/year^32,33^. In a general population, a 3.3% reduction in renal function per year corresponds to the 2.5^th^ percentile of the distribution of annual renal function loss^34^. Participants whose rate of eGFR decline was <3.5 mL/min/1.73 m^2^/year or <3.3% per year were classified as non-decliners.

Receiver operating characteristic (ROC) curve analyses were used to evaluate the diagnostic utility of plasma dp-ucMGP in distinguishing participants with DKD from participants with DM without DKD and HVs. The sample size required to identify an area under the receiver operating characteristic curve (AUC) of >0.75 with a null hypothesis AUC of 0.5, a ratio of sample sizes in negative/ positive groups of 3.34, α of 0.05 and β of 0.1 (power 90%) was determined to be 18 cases with DKD and 60 cases without. Further ROC curves were constructed to determine the utility of plasma dp-ucMGP in distinguishing participants with DKD from participants with DM without DKD and decliners from non-decliners. The relationships between natural log (LN) dp-ucMGP, and eGFR, LN uACR and change in eGFR (absolute, percentage) were explored using linear regression models and represented on scatter plots. Box and whiskers plots were used to illustrate the differences in plasma dp-ucMGP between HVs, participants with DM with and without DKD and between decliners and non-decliners (absolute, percentage).

Separate stepwise regression analyses were carried out on the HVs and the participants with DM, to determine which variables best explained the dp-ucMGP level. As dp-ucMGP was not normally distributed, the LN dp-ucMGP was used as the dependent variable. The variables listed in Table 1 (with the exception of creatinine as eGFR was included in the model) and gender (male/female) were included in the stepwise models for HVs and those with DM. As CRP, GGT, triglycerides, 25(OH) D, hsTnT, NT-proBNP and uACR were not normally distributed, the LN of each of these variables was used in the models.

## Results

**Figure 1:**
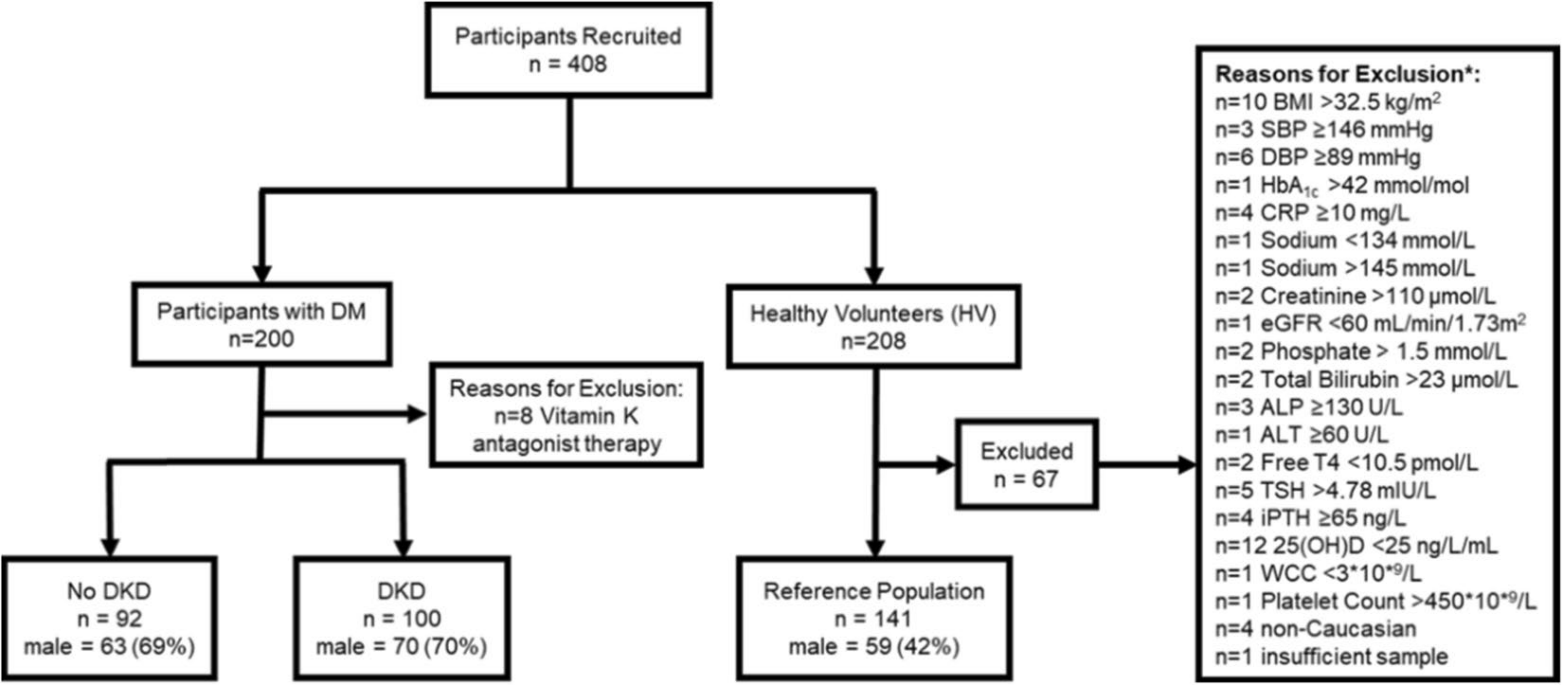
Recruitment schematic to establish normative data for dp-ucMGP in an Irish Caucasian population and to determine its clinical utility in DKD. DKD: diabetic kidney disease; BMI: body mass index; SBP: systolic blood pressure; DBP: diastolic blood pressure; HbA_1c_: glycated haemoglobin; CRP: C-reactive protein; eGFR: estimated glomerular filtration rate (CKD-EPI); ALP: alkaline phosphatase; ALT: alanine aminotransferase; T4: thyroxine; TSH: thyroid stimulating hormone; iPTH: intact parathyroid hormone; 25 (OH)D: 25-hydroxyvitamin D; WCC: white cell count. *Healthy volunteers were excluded sequentially based on a single criterion.

In total, 208 HVs were recruited to the study (Fig. 1). Of these, 67 (32.2%) were excluded based on our strict exclusion criteria, leaving a reference population of 141 metabolically healthy reference controls with normal kidney function (Table 1). Of these, 55 (39.0%) had plasma dp-ucMGP concentrations below the assay reportable range or the lower limit of quantification (300 pmol/L). Baseline characteristics of the reference population and their correlations with LN plasma dp-ucMGP are outlined in Table 1. There was no statistically significant difference in plasma dp-ucMGP between smokers (n = 13; 299 (299–472)  pmol/L) and non-smokers (n = 128; 299 (299–698) pmol/L) (p = 0.264). However, there was a significant difference in plasma dp-ucMGP between males (n = 59; 301 (299–518) pmol/L) and females (n = 82; 365 (299–698) pmol/L) (p = 0.004). Among HVs, using a stepwise regression model, increasing BMI, CRP, GGT, TSH and haemoglobin were associated with increasing plasma LN dp-ucMGP (Table 2). Interestingly, this model further suggested that males had a lower plasma LN dp-ucMGP than females (p < 0.001).

**Table 2:** Stepwise regression to determine which variables were associated with LN dp-ucMGP among healthy volunteers and participants with DM with and without DKD.

Variable	Healthy Volunteers (n = 141)			Diabetes Mellitus (n = 192)		
	Coefficient	95% CI	P-Value	Coefficient	95% CI	P-Value
Constant	4.964	(4.296, 5.632)	<0.001	3.11	(0.22, 5.99)	0.035
BMI (kg/m^2^)	0.010	(0.000, 0.020)	0.048	0.017	(0.007, 0.026)	<0.001
SBP (mmHg)				−0.004	(−0.008, −0.001)	0.017
DBP (mmHg)				0.005	(−0.001, 0.011)	0.078
HbA_1c_ (mmol/mol)	−0.012	(−0.025, 0.000)	0.059	0.003	(0.000, 0.006)	0.081
CRP (mg/L)^	0.042	(−0.001, 0.085)	0.055	0.076	(0.026, 0.125)	0.003
Sodium (mmol/L)				0.027	(0.008, 0.047)	0.006
eGFR (ml/min/1.73 m^2^)				−0.008	(−0.011, −0.006)	<0.001
Phosphate (mmol/L)				−0.24	(−0.503, 0.023)	0.074
GGT (U/L)^	0.085	(0.010, 0.16)	0.027			
Triglycerides (mmol/L)^				−0.072	(−0.170, 0.025)	0.145
TSH (mIU/L)	0.032	(−0.000, 0.064)	0.05			
iPTH (ng/L)^				0.12	(0.020, 0.219)	0.019
NT-proBNP (ng/L)^				0.057	(0.006, 0.108)	0.03
uACR (mg/mmol)^	0.03	(−0.007, 0.066)	0.111			
Haemoglobin (g/dL)	0.06	(0.019, 0.100)	0.004	−0.055	(−0.093, −0.018)	0.004
Male Gender	−0.173	(−0.265, −0.082)	<0.001	−0.101	(−0.214, 0.011)	0.079

All variables detailed in Table 1 (except creatinine) and gender were included in the model. BMI: body mass index; SBP: systolic blood pressure; DBP: diastolic blood pressure; HbA_1c_: glycated haemoglobin; CRP: C-reactive protein; eGFR: estimated glomerular filtration rate (CKD-EPI); GGT: gamma-glutamyl transferase; HDL-C: high-density lipoprotein cholesterol; T4: TSH: thyroid stimulating hormone; iPTH: intact parathyroid hormone; NT-proBNP: n-terminal pro b-type natriuretic peptide; uACR: urine albumin:creatinine ratio. ^LN of variable used in the model.

### Analytical performance of dp-ucMGP assay

Linearity of the assay was verified to a dilution of 1:6 with a recovery of 100 ± 10% (Supplementary Figure 1, Supplementary Table 1). Intra-assay precision at mean dp-ucMGP concentrations of 981 pmol/L, 4397 pmol/L and 7296 pmol/L was 4.02%, 2.28% and 2.85%, respectively (Supplementary Table 2a–c). Inter-assay precision at mean dp-ucMGP concentrations of 981 pmol/L, 4397 pmol/L and 7296 pmol/L was 5.57%, 3.25% and 3.22%, respectively (Supplementary Table 2a–c). Bias was 6.7%, 9.0% and 6.1% at dp-ucMGP concentrations of 920 pmol/L, 4033 pmol/L and 6877 pmol/L, respectively (Supplementary Table 3). Samples with a haemolytic index (HI) of 258 and 338 had 19% and 13% increases in recovery, respectively, and samples with a HI of 499 had a 9% decrease in recovery of dp-ucMGP compared to baseline (HI = 7) (Supplementary Table 4). Plasma dp-ucMGP concentrations were stable when stored at −80 °C over a 9-month period with a recovery of 100 ± 10% (Supplementary Table 5). Plasma dp-ucMGP levels were stable for 1 warm-up cycle (24 h) (recovery 100 ± 10%) and for 2 freeze-thaw cycles (recovery 100 ± 10%) (Supplementary Table 6).

### Reference intervals for plasma dp-ucMGP

**Figure 2:**
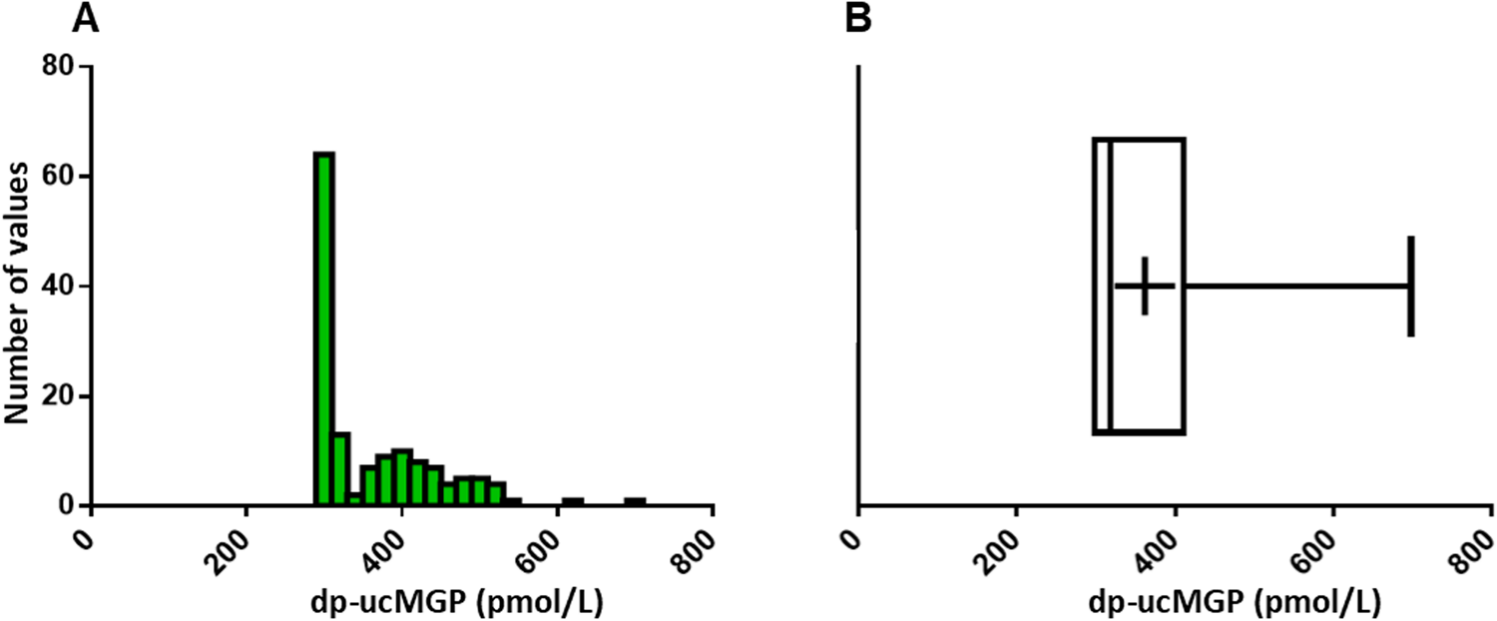
(**A**) plasma dp-ucMGP reference population histogram; (**B**) plasma dp-ucMGP reference population box and whiskers plot. The distribution of data in the histogram and box and whiskers plots clearly indicates that the values are not normally distributed. The box portion of the box and whiskers plot represents 50% of the data, the lower, median and upper quartiles. The whiskers extend to the minimum and maximum values for dp-ucMGP in the reference population. The cross represents the mean value of dp-ucMGP.

Plasma dp-ucMGP was not normally distributed among the reference population (non-Gaussian) (Fig. 2A,B). This in part was due to the proportion of HVs that had values below the LLoQ of the assay (39.0%). The D’Agostino-Pearson test rejected normality (p-value <0.001). Using the Dixon and Reed approach for detection of outliers, none were identified. Using the non-parametric method, the lower (2.5^th^ percentile) and upper (97.5^th^ percentile) reference limits for dp-ucMGP were 299 (90% CI 299–299) pmol/L and 532 (90% CI 509–698) pmol/L, respectively. As the LLoQ is 300 pmol/L, the data below 300 pmol/L is truncated. Therefore, we recommend that the RI is quoted as <300 pmol/L to 532 pmol/L.

### Diagnostic performance of plasma dp-ucMGP in DKD

**Figure 3:**
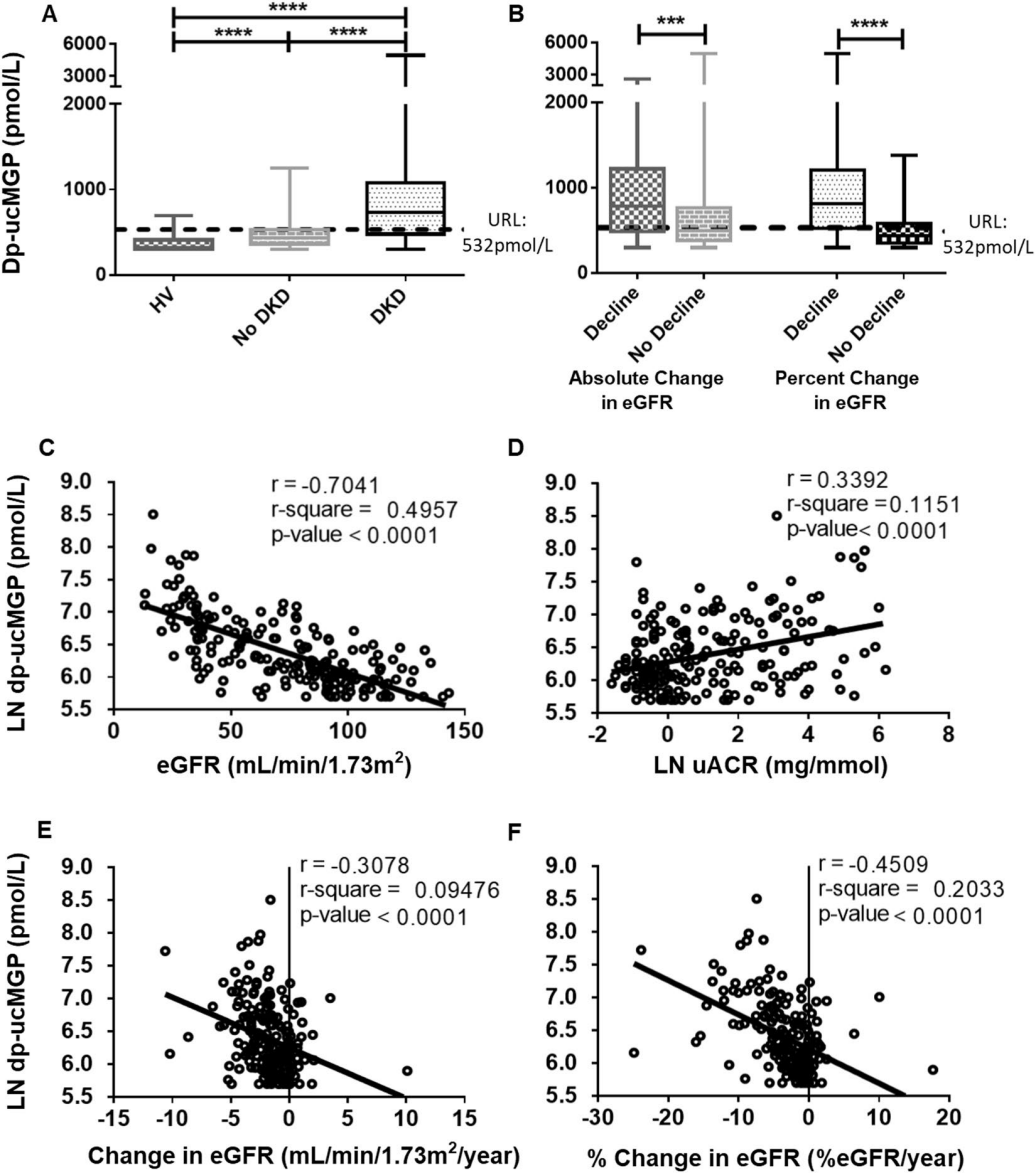
(**A**) Box and whiskers plot illustrating the levels of dp-ucMGP in HVs (n = 141), participants with DM without DKD (n = 92) and participants with DKD (n = 100). There was a stepwise increase in the level of dp-ucMGP from HVs to participants with DM without DKD to participants with DKD. Comparison between the groups is made using Kruskal Wallis with Dunn’s post-hoc for multiple comparisons. (**B**) Box and whiskers plot illustrating the levels of dp-ucMGP in decliners v non-decliners (absolute (n = 32 decliners v n = 159 non-decliners) and percentage change (n = 77 decliners v n = 114 non-decliners) in eGFR). Decliners had a higher level of dp-ucMGP compared to non-decliners. Comparison between decliners and non-decliners is made using Mann-Whitney U test. N = 1 participant did not have sufficient values available to calculate rate of decline. (**C**) Scatter plot: LN dp-ucMGP v eGFR (fitted linear regression line) for participants with DM with and without DKD. (**D**) Scatter plot: LN dp-ucMGP v LN uACR (fitted linear regression line) for participants with DM with and without DKD. (**E**) Scatter plot: LN dp-ucMGP v Change in eGFR (absolute) (fitted linear regression line) for participants with DM with and without DKD. (**F**) Scatter plot: LN dp-ucMGP v Change in eGFR (%) (fitted linear regression line) for participants with DM with and without DKD. ***p < 0.001. ****p < 0.0001.

In addition to 141 HVs, 200 participants with DM with and without DKD were recruited (Fig. 1). Of these, 8 (4%) were on Vitamin K antagonist therapy and were excluded from the study. Of the remaining 192 participants, 100 (52.1%) had DKD and 92 (47.9%) did not. Baseline biochemical, haematological and clinical parameters for the HVs, participants with DM with and without DKD are outlined in Tables 3, 4. There was a significant, stepwise increase in plasma dp-ucMGP from HVs (318 (299–698) pmol/L) to participants with DM without DKD (434 (299–1251)pmol/L) to participants with DKD (729 (299–4938) pmol/L) (p < 0.001) (Fig. 3A). A series of eGFR values, sufficient to calculate rate of change in eGFR were available for 191/192 participants with DM over a median of 7.4 (0.5–11.6) years. Based on absolute change in eGFR per year, 32 (16.8%) were categorised as decliners. However, when based on % change in eGFR per year, 77 (40.3%) were categorised as decliners. Decliners had higher plasma dp-ucMGP than non-decliners (absolute (784 (299–2611) pmol/L v 499 (299–4938) pmol/L, p < 0.001); percentage (817 (299–4938) pmol/L v 439 (299–1385) pmol/L), p < 0.001) (Fig. 3B). In patients with DM, there were significant strong negative linear associations between LN plasma dp-ucMGP and eGFR (r = −0.7041, p < 0.001), absolute change in eGFR (r = −0.3078, p < 0.001) and % change in eGFR (r = −0.4509, p < 0.001) as well as a significant, moderate positive linear association between LN plasma dp-ucMGP and LN uACR (r = 0.3392, p < 0.001) (Fig. 3C–F).

**Table 3:** Comparison of baseline clinical demographics: reference population (HV), participants with DM and no DKD and participants with DKD.

Parameter	HV	No DKD	DKD	P-value			
	n = 141	n = 92	n = 100	HV *vs* No DKD^≠^	HV *vs* DKD^≠^	No DKD *vs* DKD^≠^	Overall^¥^
Age (years)*	34.1 (12.0)	54.2 (16.8)	67.0 (13.3)	<0.001	<0.001	<0.001	<0.001
Male no. (%)~	59 (41.8)	63 (68.4)	70 (70.0)	<0.001	<0.001	0.943	<0.001
BMI (kg/m^2^)*	24.2 (3.5)	28.9 (5.8)	30.8 (6.0)	<0.001	<0.001	0.026	<0.001
Pulse (beats per min)*	70 (13)	79 (13)	79 (14)	<0.001	<0.001	0.938	<0.001
SBP (mmHg)*	122 (10)	129 (12)	137 (17)	<0.001	<0.001	<0.001	<0.001
DBP (mmHg)*	74 (7)	75 (9)	72 (11)	0.892	0.093	0.055	0.042
Smoker no. (%)~	13 (9.2)	10 (10.9)	14 (14.0)	0.851	0.341	0.662	0.506
Duration of DM (years)°	0.0 (0.0–0.0)	10.0 (0.2–59.0)	15.0 (2.0–49.0)	<0.001	<0.001	0.066	<0.001
CVD no. (%)~	0 (0.0)	7 (7.6)	30 (30.0)	N/A	N/A	<0.001	N/A
**Type of Diabetes Mellitus**^**∂**^							
Type 1 DM no. (%)	0 (0)	31 (33.7)	13 (13.0)	N/A	N/A	0.002	N/A
Type 2 DM no. (%)	0 (0)	55 (59.8)	85 (85.0)	N/A	N/A	<0.001	N/A
Other DM no. (%)	0 (0)	6 (6.5)	2 (2.0)	N/A	N/A	0.117	N/A

BMI: body mass index; SBP: systolic blood pressure; DBP: diastolic blood pressure; DM: diabetes mellitus; CVD: cardiovascular disease. *Mean (standard deviation); ~Number (percentage); ^Median (minimum to maximum). ^¥^p-values represent significance levels for multiple comparisons between the three groups – Kruskal Wallis for non-parametric data; ANOVA for parametric data; Chi-squared for frequencies. ^**≠**^Multiplicity adjusted p-values are reported for non-parametric data (Dunn’s multiple comparison), parametric data (Tukey’s multiple comparison) and frequencies (pairwise tests for independence). ^**∂**^Chi-squared to determine if there was a difference in the proportion of participants with Type 1 DM, Type 2 DM and Other DM in the DM without DKD group compared to the DKD group.

**Table 4:** Comparison of baseline biochemical and haematological parameters of the reference population (HV), participants with DM and no DKD and participants with DKD.

Parameter	HV	No DKD	DKD	P-value			
	n = 141	n = 92	n = 100	HV vs No DKD^≠^	HV vs DKD^≠^	No DKD vs DKD^≠^	Overall^¥^
dp-ucMGP (pmol/L)^	318 (299–698)	434 (299–1251)	729 (299–4938)	<0.001	<0.001	<0.001	<0.001
HbA_1c_ (mmol/mol)*	32 (3)	62 (16)	64 (17)	<0.001	<0.001	0.478	<0.001
CRP (mg/L)^	0.7 (0.5–8.9)	1.8 (0.5–46.6)	2.4 (0.5–47.3)	<0.001	<0.001	>0.999	<0.001
Sodium (mmol/L)*	140 (2)	139 (2)	139 (3)	0.003	0.003	0.996	<0.001
Potassium (mmol/L)*	4.3 (0.3)	4.4 (0.3)	4.7 (0.5)	0.156	<0.001	<0.001	<0.001
Chloride (mmol/L)*	101 (2)	99 (3)	100 (3)	<0.001	0.004	0.145	<0.001
Urea (mmol/L)*	5.0 (1.3)	5.5 (1.8)	10.3 (4.9)	0.509	<0.001	<0.001	<0.001
Creatinine (µmol/L)*	76 (13)	74 (16)	136 (60)	0.87	<0.001	<0.001	<0.001
eGFR (ml/min/1.73 m^2^)*	100 (15)	95 (20)	53 (27)	0.129	<0.001	<0.001	<0.001
Adj. Calcium (mmol/L)*	2.31 (0.06)	2.35 (0.09)	2.35 (0.08)	0.002	0.001	0.998	<0.001
Phosphate (mmol/L)*	1.12 (0.17)	1.00 (0.18)	1.08 (0.19)	<0.001	0.217	0.008	<0.001
Total Bilirubin (µmol/L)*	8.8 (3.9)	8.5 (4.9)	7.4 (6.3)	0.909	0.078	0.258	0.085
ALP (U/L)*	61 (16)	82 (22)	88 (30)	<0.001	<0.001	0.12	<0.001
ALT (U/L)*	20 (8)	26 (16)	24 (13)	0.001	0.052	0.459	0.001
GGT (U/L)*	16 (6–83)	24 (8–279)	26 (9–782)	<0.001	<0.001	0.345	<0.001
Cholesterol (mmol/L)*	4.6 (0.8)	4.1 (0.9)	4.0 (1.1)	<0.001	<0.001	0.933	<0.001
Triglycerides (mmol/L)^	0.9 (0.3–4.7)	1.3 (0.3–6.9)	1.8 (0.6–8.2)	<0.001	<0.001	0.003	<0.001
HDL-C (mmol/L)*	1.7 (0.4)	1.4 (0.5)	1.2 (0.4)	<0.001	<0.001	<0.001	<0.001
LDL-C (mmol/L)*	2.4 (0.7)	2.0 (0.7)	2.0 (0.9)	<0.001	<0.001	0.995	<0.001
FT4 (pmol/L)*	16.2 (2.2)	16.5 (3.0)	16.4 (3.1)	0.743	0.703	0.339	0.372
TSH (mIU/L)*	2.09 (0.99)	2.03 (1.13)	2.48 (1.63)	0.93	0.047	0.036	0.022
iPTH (ng/L)^	30.7 (8.1–62.3)	26.9 (10.2–90.8)	41.7 (6.3–311.1)	0.245	<0.001	<0.001	<0.001
25 (OH) D (ng/mL)	52 (25–118)	55 (14–165)	52 (14–125)	0.562	0.833	0.903	0.583
hsTnT (ng/L)*	4 (4–10)	4 (6–11)	21 (5–78)	<0.001	<0.001	<0.001	<0.001
NT-proBNP (ng/L)^	23.6 (9.0–150.0)	37.7 (9.0–811.2)	223.9 (9.0–13509.0)	0.01	<0.001	<0.001	<0.001
uACR (mg/mmol)^	0.63 (0.05–19.5)	0.7 (0.2–15.3)	10.0 (0.4–484.7)	0.89	<0.001	<0.001	<0.001
WCC (10*^9^/L)*	6.4 (1.6)	6.9 (1.9)	8.0 (2.4)	0.124	<0.001	<0.001	<0.001
Haemoglobin (g/dL)*	13.9 (1.1)	13.9 (1.3)	13.0 (1.8)	0.984	<0.001	<0.001	<0.001
Platelet Count (10*^9^/L)*	252 (51)	248 (68)	249 (85)	0.864	0.925	0.99	0.149
**Rate of Change in Renal Function**							
Absolute Change (mL/min/1.73 m^2^/year)*	N/A	−0.972 (1.363)	−2.496 (2.472)	N/A	N/A	<0.001	N/A
Percentage Change (% change/year)*	N/A	−1.179 (2.059)	−5.179 (5.478)	N/A	N/A	<0.001	N/A

Dp-ucMGP: dephosphorylated-uncarboxylated Matrix Gla-Protein; HbA_1c_: glycated haemoglobin; CRP: C-reactive protein; eGFR: estimated glomerular filtration rate (CKD-EPI); Adj. Calcium: adjusted calcium, ALP: alkaline phosphatase; ALT: alanine aminotransferase; GGT: gamma-glutamyl transferase; HDL-C: high-density lipoprotein cholesterol; LDL-C: low-density lipoprotein cholesterol; T4: thyroxine; TSH: thyroid stimulating hormone; iPTH: intact parathyroid hormone; 25 (OH) D: 25-hydroxyvitamin D; hsTnT: high-sensitivity troponin T; NT-proBNP: n-terminal pro b-type natriuretic peptide; WCC: white cell count; uACR: urine albumin:creatinine ratio. ^Median (minimum to maximum); *mean (standard deviation). ^¥^p-values represent the significance levels for multiple comparisons between the three groups – Kruskal Wallis for non-parametric data; ANOVA for parametric data. Student’s t test was used to compare rate of change (absolute, percentage) between no DKD and DKD group. ^**≠**^Multiplicity adjusted p-values are reported for non-parametric (Dunn’s multiple comparison) and parametric data (Tukey’s multiple comparison).

**Figure 4:**
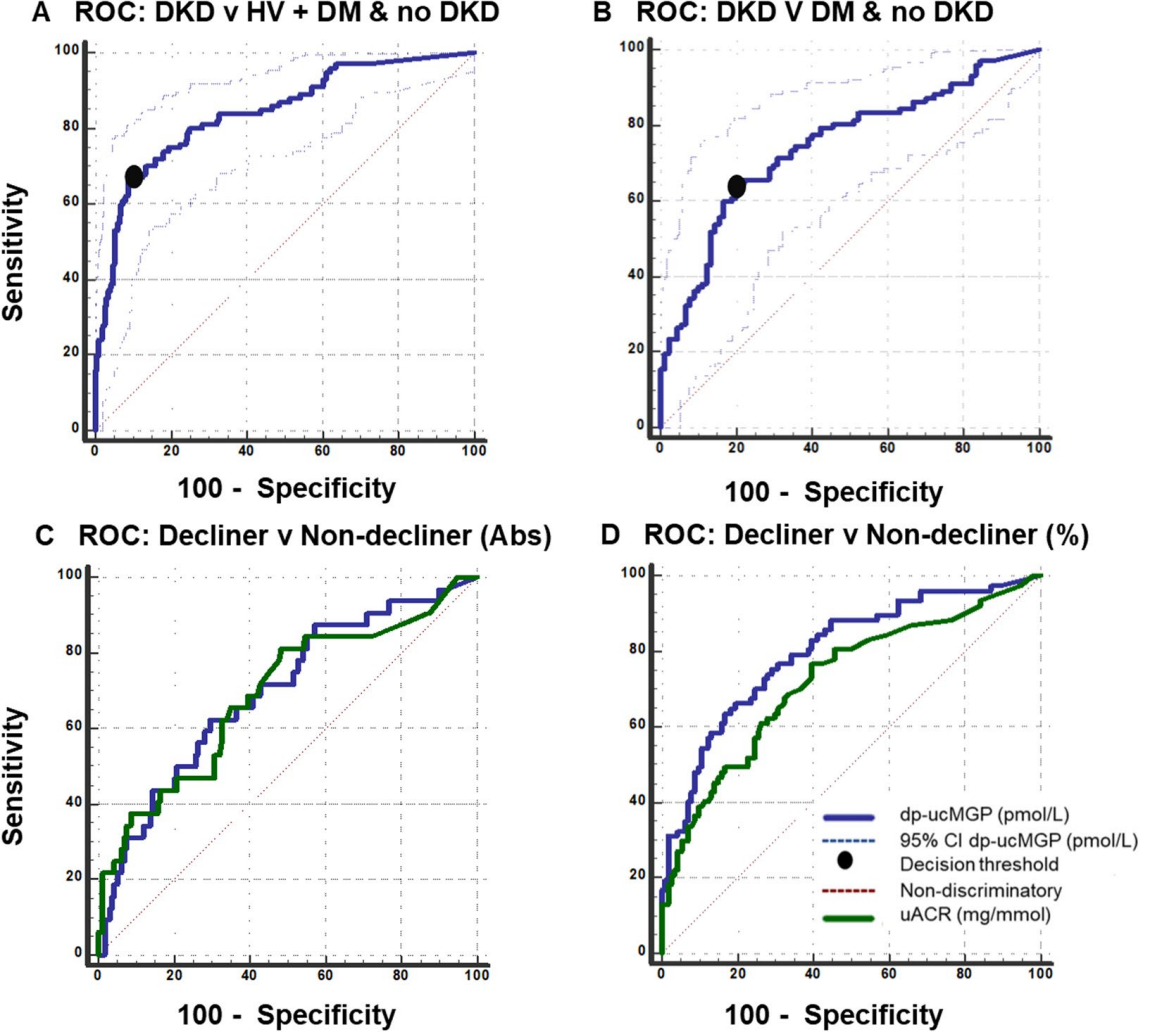
(**A**) ROC curve for dp-ucMGP: DKD (n = 100) versus DM without DKD (n = 92) and HV (n = 144). The AUC for dp-ucMGP was 0.842 (95% CI: 0.799–0.880; p < 0.001) with a diagnostic sensitivity of 67.0% (95% CI 56.9–76.1), a diagnostic specificity of 91% (95% CI 86.1–94.0), positive likelihood ratio of 7.10 (95% CI 4.7–10.8), a negative likelihood ratio of 0.36 (95% CI 0.3–0.5) and decision threshold of >557 pmol/L. (**B**) ROC curve for dp-ucMGP: DKD (n = 100) versus DM without DKD (n = 92). The AUC for dp-ucMGP was 0.747 (95% CI: 0.679–0.807; p < 0.001) with a diagnostic sensitivity of 64.7% (95% CI 54.6–73.9), a diagnostic specificity of 80.0% (95% CI 70.2–87.7), positive likelihood ratio of 3.24 (95% CI 2.1–5.0), a negative likelihood ratio of 0.44 (95% CI 0.3–0.6) and decision threshold of >570 pmol/L. (**C**) ROC curve for dp-ucMGP v uACR: decliners (n = 32) and non-decliners (n = 159) (absolute change in renal function). The AUC for dp-ucMGP was 0.696 (95% CI: 0.625–0.760, p < 0.001) compared to an AUC for uACR of 0.689 (95% CI: 0.618–0.754, p < 0.001) (p = 0.921). (**D**) ROC curve for dp-ucMGP v uACR: decliners (n = 77) and non-decliners (n = 114) (% change in renal function). The AUC for dp-ucMGP was 0.800 (95% CI: 0.737–0.853, p < 0.001) compared to an AUC for uACR of 0.723 (95% CI: 0.654–0.786, p < 0.001) (p = 0.103). The area under the ROC curve (AUC) is used to assess test accuracy. A ROC curve is constructed from sensitivity compared to 100-specificity. An AUC >0.9 is indicative of a very accurate test.

ROC curve analyses were used to determine the ability of plasma dp-ucMGP to identify participants with DKD. First, participants with DKD were compared to participants with DM without DKD and HV (Fig. 4A). This analysis produced an AUC of 0.842 (95% CI: 0.799–0.880; p < 0.001) with a diagnostic sensitivity of 67.0% (95% CI 56.9–76.1), a diagnostic specificity of 91% (95% CI 86.1–94.0), positive likelihood ratio of 7.10 (95% CI 4.7–10.8) and a negative likelihood ratio of 0.36 (95% CI 0.3–0.5). The optimum cut-off for identifying participants with DKD was a plasma dp-ucMGP concentration >557 pmol/L. Further ROC curve analysis compared participants with DKD to participants with DM without DKD (Fig. 4B). The AUC for dp-ucMGP was 0.747 (95% CI: 0.679–0.807; p < 0.001) with a diagnostic sensitivity of 64.7% (95% CI 54.6–73.9), a diagnostic specificity of 80.0% (95% CI 70.2–87.7), a positive likelihood ratio of 3.24 (95% CI 2.1–5.0), a negative likelihood ratio of 0.44 (95% CI 0.3–0.6) and decision threshold of >570 pmol/L.

ROC curve analyses were also used to compare the ability of dp-ucMGP and uACR to distinguish participants with DM with and without decline in renal function. The clinical characteristics of participants classified as decliners (with decline classified as absolute rate of change in eGFR of ≤−3.5 mL/min/1.73 m^2^/year or a % change in eGFR of ≤−3.3%/year) and non-decliners are outlined in Supplementary Tables 7 and 8. Plasma dp-ucMGP and uACR had moderate ability to distinguish decliners from non-decliners when absolute rate of change in eGFR (≤−3.5 mL/min/1.73 m^2^/year) was used (AUC of 0.696 (95% CI: 0.625–0.760, p < 0.001) versus 0.689 (95% CI: 0.618–0.754, p < 0.001) (p = 0.921); Fig. 4C). Similar findings were observed using lower and higher thresholds to classify decliner status. Using a threshold of ≤−4.5 mL/min/1.73 m^2^, the predictive value of plasma dp-ucMGP was weaker and uACR stronger; but there was no significant difference between the two indices (AUC of 0.627 (95% CI: 0.554–0.696, p = 0.131) versus 0.751 (95% CI: 0.683–0.810, p < 0.001) (p = 0.231)). Using a threshold of ≤−2.5 mL/min/1.73 m^2^, the predictive values of plasma dp-ucMGP and uACR were similar (AUC of 0.686 (95% CI: 0.615–0.751, p < 0.001) and 0.690 (95% CI: 0.619–0.755, p < 0.001) (p = 0.948).

However, when decliners were defined based on % change in eGFR (≤−3.3%/year), plasma dp-ucMGP and uACR had greater predictive values but there was no significant difference between the two indices (AUC of 0.800 (95% CI: 0.737–0.853, p < 0.001) versus 0.723 (95% CI: 0.654–0.786, p < 0.001), (p = 0.103) (Fig. 4D). Similar findings were observed using lower and higher thresholds to classify decliner status. Using a threshold of ≤−4% decline in renal function per year, the predictive values of both plasma dp-ucMGP and uACR were similiar (AUC of 0.818 (95% CI: 0.755–0.870, p < 0.001) and 0.745 (95% CI: 0.677–0.805, p < 0.001), (p = 0.150). Using a threshold of ≤−2% decline, the predictive values of both plasma dp-ucMGP and uACR were again similar (AUC of 0.756 (95% CI: 0.689–0.816, p < 0.001) versus 0.708 (95% CI: 0.638–0.772, p < 0.001), (p = 0.308).

Similar to HVs, using a stepwise regression model (Table 2) in participants with DM, increasing BMI and CRP were associated with increasing LN dp-ucMGP. Interestingly, decreasing haemoglobin was associated with increasing plasma LN dp-ucMGP – the converse was true for HVs. Other significant explanatory variables included SBP, sodium, eGFR, iPTH and NT-proBNP.

## Discussion

The quality of reference intervals for a clinical laboratory assay are as important as the quality of the result itself. Reference intervals are the decision support tools used to guide clinicians in the correct interpretation of results, helping to discriminate between those with and those without disease. Prior to the introduction of a biomarker into routine clinical practice, it is essential to establish RIs in a healthy local population. This is the first study that defined robust RIs for plasma dp-ucMGP using the IDS®-iSYS Ina Ktif (dp-ucMGP) assay in a metabolically healthy Caucasian population with normal kidney function. It provides novel insights on the utility of plasma dp-ucMGP as a biomarker of both DKD and renal function change in adults with DM. Our results demonstrated that plasma dp-ucMGP was higher in persons with DKD compared to those with DM without DKD and HV and in decliners compared to non-decliners. We also found that plasma dp-ucMGP was a measurable indicator of DKD and renal function decline. Significant negative linear relationships existed between LN plasma dp-ucMGP and eGFR as well as rate of change in renal function and a significant positive linear relationship existed between dp-ucMGP and uACR in participants with DM with and without DKD.

In previous comparable studies, Cranenburg *et al*. reported reference values for dp-ucMGP measured using dual-antibody ELISA of 477 ± 199 pmol/L in a healthy reference population (n = 75)^35^. In our study, the median value for dp-ucMGP measured using a different platform was 318 pmol/L among HVs and 434 pmol/L among those with DM without DKD. In contrast to our study, the authors noted that as age increased plasma dp-ucMGP levels increased. Within this cohort, however, 20% and 9% had known diagnoses of hypertension and hypercholesterolaemia, respectively, with frequencies that increased with age. As vascular calcification is linked to hypertension and hypercholesterolaemia^36,37^, the impact of these conditions on dp-ucMGP values is likely to be significant. The use of different platforms for measuring dp-ucMGP together with the prevalence of significant co-morbidities and less strict inclusion criteria for the healthy reference population may explain why dp-ucMGP levels are higher among the cohort reported by Cranenburg *et al*.^35^ compared to our HVs and participants with DM without DKD. Increasing age was also associated with increasing dp-ucMGP among a random sample of inhabitants in two regions and one city in Switzerland and in participants with T2DM. In the Swiss study, the proportion of participants with DM, hypertension, cardiovascular disease, reduced eGFR and elevated SBP, DBP, pulse wave velocity and BMI increased from the low to the medium to the high tertile of dp-ucMGP^38^. Among participants with T2DM, in a multivariable model following adjustment for ethnicity, eGFR and warfarin use, the association between age and dp-ucMGP was no longer statistically significant^17^. Thus, our strict inclusion and exclusion criteria may explain why no association was observed between age and dp-ucMGP in our study. This suggests that age per se (as opposed to medical conditions that become more prevalent with age) likely does not significantly influence plasma dp-ucMGP concentration.

We noted that plasma dp-ucMGP was higher in females than males while Cranenburg *et al*. found gender had no effect^35^. Both studies had different participant numbers and characteristics which could explain the gender differences, suggesting that additional studies are required to determine whether gender-specific RIs are necessary. It is of interest that, even in a reference population selected for metabolic health and BMI ≤ 32.5 kg/m^2^, we observed significant (albeit mild) correlations between LN plasma dp-ucMGP and BMI as well as LN CRP, eGFR and LN uACR. This suggests that vitamin K status and plasma dp-ucMGP have the potential to be important predictors of long-term vascular health among large populations. Notably, in the Longitudinal Aging Study Amsterdam (LASA), which evaluated community-dwelling older adults aged >55 years, free of CVD at baseline, vitamin K insufficiency as assessed by high plasma dp-ucMGP was associated with increased risk of CVD independent of the classical risk factors^39^. In contrast, in the Health ABC (Health, Aging, and Body Composition) Study of community-dwelling adults aged 70–79 years without CVD, Shea *et al*. observed that low circulating phylloquinone but not dp-ucMGP was associated with higher CVD risk in older adults treated for hypertension^40^.

In keeping with results published by others^3,9,10^, we observed that plasma dp-ucMGP increased as renal function deteriorated. Existing studies note that plasma dp-ucMGP levels increased progressively from CKD 2/3 to CKD 4/5 to CKD 5 on dialysis^10^ and from eGFR >90 mL/min/1.73 m^2^ to CKD 2 to CKD 3–5^9^. Unsurprisingly, plasma dp-ucMGP is higher in patients on haemodialysis compared to age-matched controls with normal renal function^41^. There are well established associations between plasma dp-ucMGP and concomitant levels of renal function across the CKD spectrum. In this study, we showed that there is also a relationship between plasma dp-ucMGP and rate of change in renal function (calculated using ≥ 2 values sampled over 7 + years) in adults with DM. Perhaps consistent with this, Wei *et* *al.* showed, in a general population after 8.9 years follow-up, that plasma dp-ucMGP increased by 23% while eGFR decreased by 4.05 mL/min/1.73 m^2^ ^42^, also indicating a time-dependent relationship. In that study, which included follow-up data from only a single time-point, baseline plasma dp-ucMGP predicted new onset microalbuminuria and eGFR < 60 mL/min/1.73 m^2^. In our study, the strength of association between rate of change in renal function and LN dp-ucMGP was stronger and the proportion of participants classified as decliners greater, when % rather than absolute change in renal function was used. This finding indicates that small absolute changes in renal function at lower eGFRs have a greater impact on dp-ucMGP levels than similar magnitude changes at higher eGFRs. Use of % rather than absolute change may be a more sensitive method of identifying decliners, although further study is required. Thus, regardless of current eGFR, renal function decline appears to associate with increased plasma dp-ucMGP. Importantly, high plasma dp-ucMGP is associated with increased CVD risk among patients with T2DM, in particular those with peripheral artery disease and heart failure^43^ and those with increasing severity of chronic heart failure^44^. High circulating levels of dp-ucMGP have also been shown to be associated with arterial stiffness after adjustment for common cardiovascular risk factors, renal function and age^38^, suggesting that interventions targeting the mechanisms underlying high plasma dp-ucMGP may be of distinct clinical benefit. In this regard, it has been proposed that vitamin K therapy has potential to slow vascular calcification^20^. In a trial of 17 haemodialysis patients, daily supplementation for 6 weeks with vitamin K2 resulted in a 27% reduction in plasma dp-ucMGP^41^. A further study of 38 patients with CKD 4/5 demonstrated a 10.7% reduction in plasma dp-ucMGP following supplementation with 90 µg of Vitamin K2 for 270 ± 12 days^45^. Additional trials are needed to evaluate the long-term impact of vitamin K therapy on dp-ucMGP, cardiovascular morbidity and mortality as renal function declines.

Plasma dp-ucMGP proved to have predictive value for participants with DKD from those with DM without DKD and HVs. Of note, when plasma dp-ucMGP was used to distinguish those with DKD from those with DM without DKD (and not HVs) there was a modest decrease in the AUC and sensitivity. In our study, DKD was diagnosed based on a “tarnished gold standard”- eGFR and uACR. Renal biopsy is considered the true “gold standard” but is not practical or necessary in all patients in clinical practice^46^ – as the information gained from biopsy does not always alter patient management. Consequently, it is possible that the prevalence of DKD may be under- or over- estimated; depending on the accuracy of our “tarnished gold standard.” The inclusion and exclusion criteria for HVs were strict and thus it is unlikely that any participants in this group had DM or any significant kidney disease which would lead to their misclassification. Misclassification of some participants with or without DKD may explain why the AUC and sensitivity of plasma dp-ucMGP decreased when HVs were removed from the analysis. Thus, the accuracy of dp-ucMGP in detection of DKD may be influenced by imperfect gold standard bias; which could make plasma dp-ucMGP appear better (same errors as tarnished gold standard) or worse (performs better than tarnished gold standard) than it truly is.

The RIs in our study were established in a healthy Northern European Caucasian population which limits their generalisability to other ethnicities. Our observation of higher plamsa dp-ucMGP in metabolically healthy females compared to males requires definitive confirmation and partitioning of the reference range according to sex. As 39.0% of the reference population had values < LLoQ, more sensitive assays are necessary to determine the clinical relevance of reporting values < 300 pmol/L. GFR was estimated using the CKD-EPI equation; it was not measured using a reference method. While plasma dp-ucMGP was found to distinguish HVs and patients with DM without DKD from those with DKD, it was also associated with rate of change in renal function. However, the absence of a validation cohort to affirm our findings is a limitation. Detailed data regarding vascular calcification were not collated as part of the study protocol, however, associations between MGP and vascular calcification are elucidated in the literature^3,17–20,38,39^. With a molecular mass of 10 kDa, the increase in dp-ucMGP as eGFR declines (both in HVs and participants with DM) could be attributed wholly or in part to reduced excretion^41^ rather than being induced by specific pathophysiological effects of DKD. Miyata *et al*. have shown that baseline eGFR negatively correlated with glomerular and tubulointerstitial MGP mRNA levels among patients recruited to the Nephrotic Syndrome Study Network (NEPTUNE). Furthermore, independently of eGFR, tubulointerstitial MGP was strongly associated with interstitial fibrosis, tubular atrophy, acute tubular injury and interstitial inflammation^47^. This together with the further association we observed between plasma dp-ucMGP and rate of loss in renal function, provides evidence that plasma dp-ucMGP among adults with DM is dictated by more than simple filtration. Nonetheless, as our study is observational in nature, conclusions regarding causality cannot be drawn.

## Conclusions

Reference intervals for plasma dp-ucMGP in a metabolically healthy Caucasian population with normal kidney function were established using the IDS-iSYS platform. Plasma dp-ucMGP was found to distinguish HVs and patients with DM without DKD from those with DKD with a good level of accuracy, a finding that may reflect the increased risk of vascular calcification that occurs as renal function declines. Plasma dp-ucMGP was also shown to be associated with rate of change in renal function among adults with DM with a wide range of current eGFR values. Interestingly, this association was stronger when change in eGFR was expressed in terms of % change per year rather than absolute change per year (ml/min/1.73 m^2^/year). Accurate definition of RIs and of the relationships between plasma dp-ucMGP concentrations and indices that reflect the severity of CKD in the setting of DM provide an important platform for integrating this robust biomarker into future studies on the role of vitamin K status and supplementation in cardiovascular health and on the role of the MGP system in renal function decline at a molecular level.

### Ethical approval

Ethical approval was granted by the Clinical Research Ethics Committee, Galway University Hospitals (Ref: C.A. 1404) and the National University of Ireland Galway, Research Ethics Committee (Ref: 16-July-05).

### Guarantor

PMOS.

## Supplementary information

Supplementary Data

## References

[1] Price PA, Faus SA, Williamson MK (1998). Warfarin causes rapid calcification of the elastic lamellae in rat arteries and heart valves. Arterioscler Thromb Vasc Biol.

[2] Schurgers LJ, Uitto J, Reutelingsperger CP (2013). Vitamin K-dependent carboxylation of matrix Gla-protein: a crucial switch to control ectopic mineralization. Trends Mol Med.

[3] Liabeuf S (2014). Vascular calcification in patients with type 2 diabetes: the involvement of matrix Gla protein. Cardiovasc Diabetol.

[4] Fraser JD, Price PA (1988). Lung, heart, and kidney express high levels of mRNA for the vitamin K-dependent matrix Gla protein. Implications for the possible functions of matrix Gla protein and for the tissue distribution of the gamma-carboxylase. J Biol Chem.

[5] Price PA, Fraser JD, Metz-Virca G (1987). Molecular cloning of matrix Gla protein: implications for substrate recognition by the vitamin K-dependent gamma-carboxylase. Proc Natl Acad Sci USA.

[6] Luo G (1997). Spontaneous calcification of arteries and cartilage in mice lacking matrix GLA protein. Nature.

[7] Munroe PB (1999). Mutations in the gene encoding the human matrix Gla protein cause Keutel syndrome. Nat Genet.

[8] Parker BD (2009). Association of kidney function and uncarboxylated matrix Gla protein: data from the Heart and Soul Study. Nephrol Dial Transplant.

[9] Puzantian H (2018). Circulating Dephospho-Uncarboxylated Matrix Gla-Protein Is Associated With Kidney Dysfunction and Arterial Stiffness. Am J Hypertens.

[10] Schurgers LJ (2010). The circulating inactive form of matrix gla protein is a surrogate marker for vascular calcification in chronic kidney disease: a preliminary report. Clin J Am Soc Nephrol.

[11] Kannel WB, McGee DL (1979). Diabetes and cardiovascular disease. The Framingham study. JAMA.

[12] Einarson TR, Acs A, Ludwig C, Panton UH (2018). Prevalence of cardiovascular disease in type 2 diabetes: a systematic literature review of scientific evidence from across the world in 2007–2017. Cardiovasc Diabetol.

[13] Afkarian M (2016). Clinical Manifestations of Kidney Disease Among US Adults With Diabetes, 1988–2014. JAMA.

[14] Wu M, Rementer C, Giachelli CM (2013). Vascular calcification: an update on mechanisms and challenges in treatment. Calcif Tissue Int.

[15] Afkarian M (2013). Kidney disease and increased mortality risk in type 2 diabetes. J Am Soc Nephrol.

[16] Boxma PY (2012). Vitamin k intake and plasma desphospho-uncarboxylated matrix Gla-protein levels in kidney transplant recipients. PLoS One.

[17] Sardana M (2017). Inactive Matrix Gla-Protein and Arterial Stiffness in Type 2 Diabetes Mellitus. Am J Hypertens.

[18] Mayer O (2016). Desphospho-uncarboxylated matrix Gla protein is associated with increased aortic stiffness in a general population. J Hum Hypertens.

[19] Cruickshank K (2002). Aortic pulse-wave velocity and its relationship to mortality in diabetes and glucose intolerance: an integrated index of vascular function?. Circulation.

[20] Lees, J. S., Chapman, F. A., Witham, M. D., Jardine, A. G. & Mark, P. B. Vitamin K status, supplementation and vascular disease: a systematic review and meta-analysis. *Heart*, 10.1136/heartjnl-2018-313955 (2018). 10.1136/heartjnl-2018-313955 30514729

[21] Griffin TP, Martin WP, Islam N, O’Brien T, Griffin MD (2016). The Promise of Mesenchymal Stem Cell Therapy for Diabetic Kidney Disease. Curr Diab Rep.

[22] Silaghi CN, Fodor D, Gheorghe SR, Craciun AM (2019). Serum total matrix Gla protein: Reference interval in healthy adults and variations in patients with vascular and osteoarticular diseases. Clin Chim Acta.

[23] Dalmeijer GW (2013). Circulating matrix Gla protein is associated with coronary artery calcification and vitamin K status in healthy women. J Nutr Biochem.

[24] Dalmeijer GW (2013). Circulating species of matrix Gla protein and the risk of vascular calcification in healthy women. Int J Cardiol.

[25] Martin WP (2017). Influence of Referral to a Combined Diabetology and Nephrology Clinic on Renal Functional Trends and Metabolic Parameters in Adults With Diabetic Kidney Disease. Mayo Clinic Proceedings: Innovations, Quality & Outcomes.

[26] Hamon SM (2019). Defining reference intervals for a serum growth differentiation factor-15 (GDF-15) assay in a Caucasian population and its potential utility in diabetic kidney disease (DKD). Clin Chem Lab Med.

[27] Levey AS (2009). A new equation to estimate glomerular filtration rate. Ann Intern Med.

[28] R: A Language and Environment for Statistical Computing (R Foundation for Statistical Computing, Vienna, Austria, https://www.R-project.org/, 2018).

[29] Dixon W (1953). Processing data for outliers. Biometrics.

[30] Reed AH, Henry RJ, Mason WB (1971). Influence of statistical method used on the resulting estimate of normal range. Clin Chem.

[31] Krolewski AS (2015). Progressive renal decline: the new paradigm of diabetic nephropathy in type 1 diabetes. Diabetes care.

[32] Krolewski AS (2014). Early progressive renal decline precedes the onset of microalbuminuria and its progression to macroalbuminuria. Diabetes Care.

[33] Ficociello LH (2010). High-normal serum uric acid increases risk of early progressive renal function loss in type 1 diabetes: results of a 6-year follow-up. Diabetes Care.

[34] Lindeman RD, Tobin J, Shock NW (1985). Longitudinal studies on the rate of decline in renal function with age. J Am Geriatr Soc.

[35] Cranenburg EC (2010). Characterisation and potential diagnostic value of circulating matrix Gla protein (MGP) species. Thromb Haemost.

[36] Kalra SS, Shanahan CM (2012). Vascular calcification and hypertension: cause and effect. Ann Med.

[37] Karwowski W, Naumnik B, Szczepanski M, Mysliwiec M (2012). The mechanism of vascular calcification - a systematic review. Med Sci Monit.

[38] Pivin E (2015). Inactive Matrix Gla-Protein Is Associated With Arterial Stiffness in an Adult Population-Based Study. Hypertension.

[39] van den Heuvel EG (2014). Circulating uncarboxylated matrix Gla protein, a marker of vitamin K status, as a risk factor of cardiovascular disease. Maturitas.

[40] Shea MK (2017). Circulating Vitamin K Is Inversely Associated with Incident Cardiovascular Disease Risk among Those Treated for Hypertension in the Health, Aging, and Body Composition Study (Health ABC). J Nutr.

[41] Schlieper G (2011). Circulating nonphosphorylated carboxylated matrix gla protein predicts survival in ESRD. J Am Soc Nephrol.

[42] Wei FF (2018). Desphospho-uncarboxylated matrix Gla protein is a novel circulating biomarker predicting deterioration of renal function in the general population. Nephrol Dial Transplant.

[43] Dalmeijer GW (2013). Matrix Gla protein species and risk of cardiovascular events in type 2 diabetic patients. Diabetes Care.

[44] Ueland T (2011). Circulating levels of non-phosphorylated undercarboxylated matrix Gla protein are associated with disease severity in patients with chronic heart failure. Clin Sci (Lond).

[45] Kurnatowska I (2016). Plasma Desphospho-Uncarboxylated Matrix Gla Protein as a Marker of Kidney Damage and Cardiovascular Risk in Advanced Stage of Chronic Kidney Disease. Kidney Blood Press Res.

[46] Biesenbach G, Bodlaj G, Pieringer H, Sedlak M (2011). Clinical versus histological diagnosis of diabetic nephropathy–is renal biopsy required in type 2 diabetic patients with renal disease?. QJM.

[47] Miyata KN (2018). Renal matrix Gla protein expression increases progressively with CKD and predicts renal outcome. Exp Mol Pathol.

