# Supplementary material for: Plasma dephosphorylated-uncarboxylated Matrix Gla-Protein (dp-ucMGP): reference intervals in Caucasian adults and diabetic kidney disease biomarker potential
Source: Sci Rep. 2019 Dec 5;9:18452. doi: 10.1038/s41598-019-54762-2 (PMC6895103; doi:10.1038/s41598-019-54762-2)
Supplement: Supplementary file 1 — Supplementary Data [file 41598_2019_54762_MOESM1_ESM.docx]

**Title:** **Plasma dephosphorylated-uncarboxylated Matrix Gla-Protein (dp-ucMGP): reference intervals in Caucasian adults and diabetic kidney disease biomarker potential.**

^∞^Tomás Patrick Griffin^1,2^,

^∞^Md Nahidul Islam ^2,3^,

Deirdre Wall^4^,

John Ferguson^5^,

Damian Gerard Griffin^3^,

^Matthew Dallas Griffin^2,6^,

*^Paula M O’Shea^3^

1. Centre for Endocrinology, Diabetes and Metabolism, Saolta University Health Care Group (SUHCG), Galway University Hospitals, Galway, Ireland.
2. Regenerative Medicine Institute (REMEDI) at CÚRAM SFI Research Centre, School of Medicine, National University of Ireland Galway, Galway, Ireland.
3. Department of Clinical Biochemistry, Saolta University Health Care Group, Galway University Hospitals, Galway, Ireland.
4. School of Mathematics, Statistics and Applied Mathematics, National University of Ireland Galway, Galway, Ireland.
5. Health Research Board (HRB), Clinical Research Facility, National University of Ireland Galway, Galway, Ireland.
6. Department of Nephrology, Saolta University Health Care Group, Galway University Hospitals, Galway, Ireland.

^∞^^ contributed equally to this work

**Corresponding author:**

Dr Paula M. O’Shea,

Department of Clinical Biochemistry, Saolta University Health Care Group (SUHCG), Galway University Hospitals, Newcastle Road, Galway, Ireland.

Email: [PaulaM.OShea@hse.ie](mailto:PaulaM.OShea@hse.ie).

Telephone: +353(0)91 544000

**Supplementary Methodology 1:**

**Data collection:**

The reference population and participants with DM and CKD had baseline demographics (age, gender and smoking status) and clinical characteristics recorded. Weight was measured in kilograms using the Tanita® scale and height in metres using a Seca® wall-mounted stadiometer, in accordance with departmental standard operating procedures. Pulse rate was recorded at the radial artery in the dominant hand as beats per minute.

**Supplementary Methodology 2:**

**Laboratory Sampling Strategy:**

HbA_1c_ was measured using high-performance liquid chromatography (Menarini® HA8160 auto-analyzer). Serum creatinine was measured using the isotope dilution mass spectrometry (IDMS)-traceable creatinase assay. Serum electrolytes and lipids were measured using conventional Roche Diagnostics assays. LDL-C was calculated using the Friedewald equation [1]. BN II nephelometer (Siemens Medical Solutions) was used to measure uACR. All assays were performed in the Clinical Biochemistry/Haematology/Immunology Departments, GUH.

**Supplementary Methodology 3:**

**Assessment of assay performance specifications:**

*Linearity:*

A dilution study was performed to determine assay linearity. Samples were prepared by diluting a known high participant sample (7,187 pmol/L) with IDS-iSYS Diluent A (IS-10DA). The dp-ucMGP concentration in the IS-10DA was measured and found to be below the Lower Limit of Quantification (LLoQ) (<300pmol/L). Samples were run in duplicate, with the mean observed dp-ucMGP concentration and the % recovery calculated and expected values calculated. The observed versus the expected dp-ucMGP values were represented on a scatter plot. The relationship between both values was explored using a linear regression model.

*Precision and Bias:*

As no reference material for assessment of plasma dp-ucMGP was readily available during the study period, precision and bias were assessed using the Kit Controls (IS-4730). The manufacturer-assigned controls had the following concentrations: control 1: 920pmol/L (CTRL Lot- #3428); control 2: 4033 pmol/L (CTRL Lot- #3429); control 3: 6877 pmol/L (CTRL Lot- #3430). For assessment of precision, dp-ucMGP was measured on 5 consecutive days in quintuplicate at each control level. For assessment of bias, the mean of the quintuplicate measurements on the 5 consecutive days for each level of the control were compared [2].

*Effect of haemolysis on plasma dp-ucMGP measurement:*

The effect of haemolysis was determined using whole blood from a single participant collected in two EDTA (plasma) specimen tubes. One specimen tube underwent vigorous vortexing to ensure red cell rupture and consequent haemolysis (haem). Plasma was separated from the second tube (neat) – as previously described. Samples were prepared for measurement using different ratios of haem to neat. Haemolytic index (HI) and plasma dp-ucMGP measurement were performed on each sample. The dp-ucMGP values were compared between the haemolysed and non-haemolysed samples.

*Effect of sample stability on plasma dp-ucMGP measurement:*

Plasma dp-ucMGP was measured using samples from eight participants at two time points (9 months apart) from 2 separate aliquots. A range of concentrations (299pmol/L to 4773pmol/L) were assessed. Each aliquot, stored at -80°C, was thawed for the first time prior to dp-ucMGP measurement.

*Effect of repeated freeze-thaw cycles on plasma dp-ucMGP measurement:*

The effects of warm-up and freeze-thaw cycles on plasma dp-ucMGP were evaluated using plasma samples from two separate HVs. In the case of both the warm-up and freeze-thaw cycles, plasma samples had dp-ucMGP measured at baseline. Samples were stored either in a fridge at 4°C (warm-up cycle) or in a -80°C freezer (freeze-thaw cycle). The respective samples were then removed from the fridge/freezer every 24 hours, placed on a roller to ensure adequate mixing and allowed to reach room temperature over 30 minutes. Plasma dp-ucMGP was measured and the samples returned to either the 4°C fridge or -80°C freezer. Dp-ucMGP concentration was measured every 24 hours for 4 days in both samples. Dp-ucMGP concentration was again measured after storage at -80°C for 9 months.

**Supplementary Methodology 4:**

**Statistical analyses:**

Outlier removal was based on the difference between the most extreme observation and the closest measured observation (D), divided by the range of all observations including the extreme (R) – D/R ratio. In accordance with the International Federation of Clinical Chemistry and Laboratory Medicine (IFCC) working group, an outlier was excluded when D was ≥one third R. The IFCC working group [3] supports this approach to statistically significant outliers.

**Supplementary Figure 1: Evaluation of Linearity of plasma dp-ucMGP**

**Supplementary Table 1:** **Evaluation of Linearity of plasma dp-ucMGP**

| **Sample Preparation** | | **dp-ucMGP concentration (pmol/L)** | | | | **% Recovery** |
| --- | --- | --- | --- | --- | --- | --- |
|  |  | **Replicate 1** | **Replicate 2** | **Observed** | **Expected** |  |
| **Blank** | Diluent | <300 | <300 | <300 | <300 | 100 |
| **Neat** | High Control | 7357 | 7018 | 7187 | 7187 | 100 |
| **3+1** | 300µl neat + 100µl dil | 5407 | 5302 | 5355 | 5390 | 99 |
| **1+1** | 200µl neat + 200µl dil | 3596 | 3563 | 3579 | 3594 | 100 |
| **1+2** | 250µl neat + 500µl dil | 2430 | 2460 | 2445 | 2396 | 102 |
| **1+4** | 100µl neat + 400µl dil | 1489 | 1441 | 1465 | 1437 | 102 |
| **1+6** | 100µl neat + 600µl dil | 1074 | 1081 | 1078 | 1027 | 105 |
| **1+9** | 100µl neat + 900µl dil | 829 | 801 | 815 | 719 | 113 |
| **1+19** | 100µl neat + 1900µl dil | 466 | 431 | 448 | 359 | 125 |

Samples were prepared by diluting a high sample (7,187 pmol/L) with IDS-iSYS Diluent A (IS-10DA). The dp-ucMGP concentration in the IS-10DA was measured and found to be below the LoQ (<300pmol/L). Samples were run in duplicate, with the mean observed dp-ucMGP concentration and the % recovery calculated. The observed versus the expected dp-ucMGP were represented on a scatter plot. The relationship between the observed and expected dp-ucMGP was explored using a linear regression model.

Supplementary Table 2a: Assessment of precision of the IDS-iSYS Ina*K*tif MGP (dp-ucMGP) – Low dp-ucMGP concentration

|  | **Level 1** | | | | | | | |
| --- | --- | --- | --- | --- | --- | --- | --- | --- |
|  | **Replicate 1 pmol/L** | **Replicate 2 pmol/L** | **Replicate 3 pmol/L** | **Replicate 4 pmol/L** | **Replicate 5 pmol/L** | **Run Mean pmol/L** | **Run SD (pmol/L)** | **Variance pmol/L** |
| **Day 1** | 902 | 917 | 950 | 948 | 994 | 942 | 35 | 1006 |
| **Day 2** | 912 | 910 | 898 | 951 | 978 | 930 | 34 | 899 |
| **Day 3** | 965 | 992 | 1105 | 1065 | 982 | 1022 | 60 | 2897 |
| **Day 4** | 1003 | 1092 | 983 | 955 | 982 | 1003 | 53 | 2213 |
| **Day 5** | 1001 | 1022 | 1021 | 988 | 1021 | 1011 | 15 | 190 |
|  | N | | 25 |  |  |  |  |  |
|  | Overall Mean (pmol/L) | | 981 |  |  |  |  |  |
|  | Within run variance (Vr) (pmol/L) | | 1441 |  |  |  |  |  |
|  | Within run SD (pmol/L) | | 39 |  |  |  |  |  |
|  | Within run CV% | | 4.02 |  |  |  |  |  |
|  | Between run SD (pmol/L) | | 42 |  |  |  |  |  |
|  | Between run variance (Vb) (pmol/L) | | 1430 |  |  |  |  |  |
|  | Ratio Vr/Vb | | 1.01 |  |  |  |  |  |
|  | Total variance (pmol/L) | | 2871 |  |  |  |  |  |
|  | Laboratory SD | | 55 |  |  |  |  |  |
|  | Laboratory CV% | | 5.57 |  |  |  |  |  |

Precision was assessed using Kit Controls (IS-4730). The manufacturer assigned the controls the following values: control 1: 920pmol/L (CTRL Lot- #3428); control 2: 4033 pmol/L (CTRL Lot- #3429); control 3: 6877 pmol/L (CTRL Lot- #3430).

Supplementary Table 2b: Assessment of precision of the IDS-iSYS Ina*K*tif MGP (dp-ucMGP) – Medium dp-ucMGP concentration

|  | **Level 2** | | | | | | | | |
| --- | --- | --- | --- | --- | --- | --- | --- | --- | --- |
|  | **Replicate 1 pmol/L** | **Replicate 2 pmol/L** | **Replicate 3 pmol/L** | **Replicate 4 pmol/L** | **Replicate 5 pmol/L** | **Run Mean pmol/L** | **Run SD (pmol/L)** | **Variance pmol/L** |  |
| **Day 1** | 4306 | 4513 | 4489 | 4656 | 4560 | 4505 | 128 | 13149 |  |
| **Day 2** | 4121 | 4220 | 4211 | 4391 | 4293 | 4247 | 101 | 8151 |  |
| **Day 3** | 4329 | 4331 | 4328 | 4421 | 4414 | 4365 | 48 | 1871 |  |
| **Day 4** | 4440 | 4510 | 4406 | 4613 | 4660 | 4526 | 109 | 9515 |  |
| **Day 5** | 4237 | 4287 | 4259 | 4504 | 4418 | 4341 | 115 | 10591 |  |
|  | N | | 25 |  |  |  |  |  |  |
|  | Overall Mean (pmol/L) | | 4397 |  |  |  |  |  |  |
|  | Within run variance (Vr) (pmol/L) | | 8656 |  |  |  |  |  |  |
|  | Within run SD (pmol/L) | | 100 |  |  |  |  |  |  |
|  | Within run CV% | | 2.28 |  |  |  |  |  |  |
|  | Between run SD (pmol/L) | | 117 |  |  |  |  |  |  |
|  | Between run variance (Vb) (pmol/L) | | 10967 |  |  |  |  |  |  |
|  | Ratio Vr/Vb | | 0.79 |  |  |  |  |  |  |
|  | Total variance (pmol/L) | | 19623 |  |  |  |  |  |  |
|  | Laboratory SD | | 143 |  |  |  |  |  |  |
|  | Laboratory CV% | | 3.25 |  |  |  |  |  |  |

Precision was assessed using Kit Controls (IS-4730). The manufacturer assigned the controls the following values: control 1: 920pmol/L (CTRL Lot- #3428); control 2: 4033 pmol/L (CTRL Lot- #3429); control 3: 6877 pmol/L (CTRL Lot- #3430).

Supplementary Table 2c: Assessment of precision of the IDS-iSYS Ina*K*tif MGP (dp-ucMGP) – High dp-ucMGP concentration

|  | **Level 3** | | | | | | | | |
| --- | --- | --- | --- | --- | --- | --- | --- | --- | --- |
|  | **Replicate 1 pmol/L** | **Replicate 2 pmol/L** | **Replicate 3 pmol/L** | **Replicate 4 pmol/L** | **Replicate 5 pmol/L** | **Run Mean pmol/L** | **Run SD (pmol/L)** | **Variance pmol/L** |  |
| **Day 1** | 6942 | 7233 | 7236 | 7210 | 7359 | 7196 | 154 | 18850 |  |
| **Day 2** | 7130 | 7132 | 7169 | 7275 | 7217 | 7185 | 62 | 3043 |  |
| **Day 3** | 6980 | 7174 | 7409 | 7549 | 7428 | 7308 | 228 | 41644 |  |
| **Day 4** | 7420 | 6972 | 7421 | 7758 | 7830 | 7480 | 341 | 92986 |  |
| **Day 5** | 6958 | 7234 | 7234 | 7555 | 7566 | 7309 | 255 | 52203 |  |
|  | N | | 25 |  |  |  |  |  |  |
|  | Overall Mean (pmol/L) | | 7296 |  |  |  |  |  |  |
|  | Within run variance (Vr) (pmol/L) | | 41745 |  |  |  |  |  |  |
|  | Within run SD (pmol/L) | | 208 |  |  |  |  |  |  |
|  | Within run CV% | | 2.85 |  |  |  |  |  |  |
|  | Between run SD (pmol/L) | | 119 |  |  |  |  |  |  |
|  | Between run variance (Vb) (pmol/L) | | 11333 |  |  |  |  |  |  |
|  | Ratio Vr/Vb | | 3.68 |  |  |  |  |  |  |
|  | Total variance (pmol/L) | | 53078 |  |  |  |  |  |  |
|  | Laboratory SD | | 235 |  |  |  |  |  |  |
|  | Laboratory CV% | | 3.22 |  |  |  |  |  |  |

Precision was assessed using Kit Controls (IS-4730). The manufacturer assigned the controls the following values: control 1: 920pmol/L (CTRL Lot- #3428); control 2: 4033 pmol/L (CTRL Lot- #3429); control 3: 6877 pmol/L (CTRL Lot- #3430).

Intra-assay precision for dp-ucMGP at mean concentrations of 981pmol/L, 4397pmol/L and 7296pmol/L were 4.02%, 2.28% and 2.85%, respectively. Inter-assay precision at mean dp-ucMGP concentrations of 981pmol/L, 4397pmol/L and 7296pmol/L were 5.57%, 3.25% and 3.22%, respectively, and were in accord with the claims of the manufacturer (8.2%, 3.4%, 3.3%).

**Supplementary Table 3: Assessment of bias of the IDS-iSYS Ina*K*tif MGP (dp-ucMGP)**

| **Material** | **Replicate 1 pmol/L** | **Replicate 2 pmol/L** | **Replicate 3 pmol/L** | **Replicate 4 pmol/L** | **Replicate 5 pmol/L** | **Mean pmol/L** | **Assigned pmol/L** | **% Bias** |
| --- | --- | --- | --- | --- | --- | --- | --- | --- |
| **1** | 942 | 930 | 1022 | 1003 | 1011 | 982 | 920 | 6.7% |
| **2** | 4505 | 4247 | 4365 | 4526 | 4341 | 4397 | 4033 | 9.0% |
| **3** | 7196 | 7185 | 7308 | 7480 | 7309 | 7296 | 6877 | 6.1% |

Bias was assessed using Kit Controls (IS-4730). The manufacturer assigned the controls the following values: control 1: 920pmol/L (CTRL Lot- #3428); control 2: 4033 pmol/L (CTRL Lot- #3429); control 3: 6877 pmol/L (CTRL Lot- #3430). Bias was 6.7%, 9.0% and 6.1% at 920pmol/L, 4033pmol/L and 6877pmol/L, respectively.

**Supplementary Table 4: Effect of haemolysis on plasma dp-ucMGP concentrations**

| **Sample Preparation** | | **Haemolytic Index** | **dp-ucMGP (pmol/L)** | **Recovery** |
| --- | --- | --- | --- | --- |
| **Plasma Dilutions** | |  |  |  |
| **0+1** | **0µl haem + 1000µl neat** | 7 | 416 | 100% |
| **1+1** | **500µl haem + 500µl neat** | 258 | 494 | 119% |
| **2+1** | **1000µl haem + 500µl neat** | 338 | 472 | 113% |
| **1+0** | **1000µl haem + 0µl neat** | 499 | 380 | 91% |

The effect of haemolysis was determined using whole blood from a single healthy volunteer collected in two EDTA (plasma) specimen tubes. One specimen tube underwent vigorous vortexing to ensure red cell rupture and consequent haemolysis (haem). Plasma was separated from the second tube. The EDTA vacutainer was centrifuged at 3000*g for 10 minutes at 4°C (neat). Samples were prepared for measurement using different ratios of haem to neat. Haemolytic index (HI) and dp-ucMGP measurements were performed. The effect of haemolysis on assay measurement of dp-ucMGP concentration was variable. Samples with a haemolytic index (HI) of 258 and 338 had a 19% and 13% increase in recovery, respectively, and samples with a HI of 499 had a 9% decrease in recovery of dp-ucMGP compared to baseline (HI=7).

| **Sample** | **Time point** | | **% Difference between**  **Time 0 and 9 month(s)** |
| --- | --- | --- | --- |
|  | **0 month**  **Result pmol/L** | **9 months**  **Result pmol/L** |  |
| **A** | 299 | 299 | 0% |
| **B** | 455 | 413 | 9% |
| **C** | 555 | 567 | 2% |
| **D** | 458 | 446 | 3% |
| **E** | 799 | 839 | 5% |
| **F** | 1454 | 1499 | 3% |
| **G** | 3595 | 3705 | 3% |
| **H** | 4773 | 5652 | 18% |

**Supplementary Table 5: Plasma dp-ucMGP sample stability over time**

Dp-ucMGP was measured from eight participants at two different time points from 2 separate aliquots. Each aliquot was thawed for the first time prior to these measurements. The aliquots were stored at -80°C prior to analyses. Plasma dp-ucMGP concentrations were stable when stored at -80°C with a recovery of 100 ± 10%.

| **Time Point** | **Storage Condition** | | | |
| --- | --- | --- | --- | --- |
|  | **4°C** | | **-80°C** | |
|  | ***dp-ucMGP (pmol/L)*** | ***% Recovery*** | ***dp-ucMGP (pmol/L)*** | ***% Recovery*** |
| **Day 0** | 403 | N/A | 520 | N/A |
| **Day 1** | 362 | 90% | 472 | 91% |
| **Day 2** | 346 | 86% | 497 | 96% |
| **Day 3** | 340 | 84% | 455 | 88% |
| **Day 4** | 299 | 74% | 428 | 82% |
| **Month 9** | N/A | N/A | 443 | 85% |

**Supplementary Table 6: Effect of warm-up and freeze-thaw cycles on plasma dp-ucMGP**

The effect of warm-up and freeze-thaw cycles on plasma dp-ucMGP was evaluated using plasma samples from two separate HVs. In the case of both the warm-up and freeze-thaw cycles, plasma samples had dp-ucMGP measured at baseline. Samples were stored either in a fridge at 4°C (warm-up cycle) or in a -80°C freezer (freeze-thaw cycle), removed from the fridge/freezer every 24 hours, placed on a roller to ensure adequate mixing and allowed to reach room temperature over 30 minutes. Dp-ucMGP was measured and the samples returned to either the 4°C fridge or -80°C freezer. Dp-ucMGP was measured every 24 hours for 4 days in both samples. Dp-ucMGP was again measured after 9 months storage at -80°C. Following warm up cycles, dp-ucMGP levels were stable for 1 cycle (recovery 100 ± 10%). Following freeze-thaw cycles, dp-ucMGP levels were stable for 2 cycles (recovery 100 ± 10%).

| **Parameter** | **Absolute Change in Renal Function** | | | **Percentage Change in Renal Function** | | |
| --- | --- | --- | --- | --- | --- | --- |
|  | **Decline** | **No-Decline** | **P-Value**^¥^ | **Decline** | **No-Decline** | **P-Value**^¥^ |
|  | **n=32** | **n=159** |  | **n=77** | **n=114** |  |
| Age (years)* | 64.4 (15.2) | 60.1 (16.6) | 0.17 | 68.0 (12.8) | 55.9 (16.8) | <0.001 |
| Male no. (%)~ | 24 (75.0) | 108 (67.9) | 0.429 | 53 (68.8) | 79 (69.3) | 0.945 |
| BMI (kg/m^2^)* | 30.3 (6.2) | 29.8 (5.9) | 0.692 | 30.9 (5.7) | 29.2 (6.1) | 0.066 |
| Pulse (beats per min)* | 80 (14) | 79 (14) | 0.588 | 77 (14) | 81 (13) | 0.069 |
| SBP (mmHg)* | 135 (15) | 133 (16) | 0.358 | 135 (16) | 132 (15) | 0.275 |
| DBP (mmHg)* | 72 (10) | 73 (10) | 0.425 | 70 (10) | 75 (10) | 0.002 |
| Smoker no. (%)~ | 7 (21.9) | 16 (10.1) | 0.061 | 9 (11.7) | 14 (12.3) | 0.902 |
| Duration of DM (years)° | 11.3 (2.0 - 40.0) | 12.0 (0.2 - 59.0) | 0.927 | 15.0 (1.0 - 41.0) | 11.0 (0.2 - 59.0) | 0.048 |
| CVD no. (%)~ | 11 (34.4) | 26 (16.4) | 0.019 | 25 (32.5) | 12 (10.5) | <0.001 |
| **Type of Diabetes Mellitus^∂^** | | | | | | |
| Type 1 DM no. (%) | 8 (25.0) | 36 (22.6) | 0.773 | 11 (14.3) | 33 (28.9) | 0.018 |
| Type 2 DM no. (%) | 23 (71.9) | 116 (73.0) | 0.9 | 64 (83.1) | 75 (65.8) | 0.008 |
| Other DM no. (%) | 1 (3.1) | 7 (4.4) | 0.742 | 2 (2.6) | 6 (5.3) | 0.367 |

**Supplementary Table 7:** Comparison of baseline clinical demographics: decliners versus non-decliners classified based on absolute decline (≤-3.5mL/min/1.73m^2^/year) or percentage decline (≤-3.3%/year) in renal function.

BMI: body mass index; SBP: systolic blood pressure; DBP: diastolic blood pressure; DM: diabetes mellitus; CVD: cardiovascular disease. * Mean (standard deviation); ~ Number (percentage); ^ Median (minimum to maximum). ^¥^ p-values represent significance levels for comparisons between decline and no-decline groups – students t-test for parametric data, Mann Whitney test for non-parametric data and Chi-squared for frequencies. **^∂^** Chi-squared was used to determine if there was a difference in the proportion of participants with Type 1 DM, Type 2 DM and Other DM in the decline versus no-decline groups.

| **Parameter** | **Absolute Change in Renal Function** | | | **Percentage Change in Renal Function** | | |
| --- | --- | --- | --- | --- | --- | --- |
|  | **Decline** | **No-Decline** | **P-Value** | **Decline** | **No-Decline** | **P-Value** |
|  | **n=32** | **n=159** |  | **n=77** | **n=114** |  |
| dp-ucMGP (pmol/L)^ | 784 (299 - 2611) | 499 (299 - 4938) | <0.001 | 817 (299 - 4938) | 439 (299 - 1385) | <0.001 |
| HbA_1c_ (mmol/mol)* | 62.3 (20.6) | 62.9 (15.9) | 0.857 | 61.5 (16.9) | 63.6 (16.6) | 0.401 |
| CRP (mg/L)^ | 2.2 (0.5 - 11.2) | 2.0 (0.5 - 47.3) | 0.893 | 2.7 (0.5 - 18.2) | 1.9 (0.5 - 47.3) | 0.499 |
| Sodium (mmol/L)* | 140 (3) | 139 (3) | 0.281 | 140 (3) | 139 (3) | 0.25 |
| Potassium (mmol/L)* | 4.7 (0.6) | 4.5 (0.4) | 0.015 | 4.8 (0.5) | 4.4 (0.4) | <0.001 |
| Chloride (mmol/L)* | 101 (3) | 99 (3) | 0.007 | 100 (3) | 99 (3) | 0.004 |
| Urea (mmol/L)* | 11.4 (5.1) | 7.3 (4.0) | <0.001 | 11.4 (4.9) | 5.7 (2.0) | <0.001 |
| Creatinine (µmol/L) | 154 (67) | 97 (46) | <0.001 | 149 (59) | 78 (23) | <0.001 |
| eGFR (ml/min/1.73m^2^)* | 46 (23) | 78 (31) | <0.001 | 46 (21) | 91 (23) | <0.001 |
| Adj. Calcium (mmol/L)* | 2.32 (0.09) | 2.35 (0.08) | 0.051 | 2.35 (0.08) | 2.35 (0.08) | 0.655 |
| Phosphate (mmol/L)* | 1.09 (0.23) | 1.03 (0.18) | 0.121 | 1.07 (0.20) | 1.03 (0.18) | 0.129 |
| Total Bilirubin (µmol/L)* | 6.2 (2.7) | 8.2 (6.1) | 0.06 | 6.5 (2.4) | 8.8 (7.0) | 0.007 |
| ALP (U/L)* | 92 (38) | 84 (23) | 0.11 | 92 (31) | 81 (22) | 0.007 |
| ALT (U/L)* | 21 (12) | 25 (15) | 0.131 | 23 (11) | 26 (17) | 0.151 |
| GGT (U/L)* | 25 (9- 145) | 26 (8-782) | 0.789 | 26 (9-782) | 25 (8-279) | 0.604 |
| Cholesterol (mmol/L)* | 4.0 (1.0) | 4.1 (1.0) | 0.561 | 4.0 (1.1) | 4.1 (1.0) | 0.561 |
| Triglycerides (mmol/L)^ | 1.6 (0.7-6.3) | 1.5 (0.3-8.2) | 0.322 | 1.8 (0.5 - 8.2) | 1.4 (0.3 - 6.9) | 0.004 |
| HDL-C (mmol/L)* | 1.1 (0.3) | 1.3 (0.5) | 0.03 | 1.2 (0.4) | 1.4 (0.5) | 0.005 |
| LDL-C (mmol/L)* | 2.0 (0.9) | 2.0 (0.8) | 0.64 | 1.9 (0.8) | 2.0 (0.8) | 0.583 |
| FT4 (pmol/L)* | 17.3 (2.9) | 16.2 (3.0) | 0.062 | 16.5 (3.3) | 16.4 (2.8) | 0.866 |
| TSH (mIU/L)* | 2.2 (1.3) | 2.3 (1.5) | 0.628 | 2.38 (1.35) | 2.21 (1.48) | 0.417 |
| iPTH (ng/L)^ | 49.1 (13.8-252.4) | 31.1 (6.3 - 311.1) | <0.001 | 46.4 (11.6 - 311.1) | 27.4 (6.3 - 107.2) | <0.001 |
| 25 (OH) D (ng/mL)^ | 39 (14 - 120) | 55 (14 - 165) | 0.018 | 50 (14 - 120) | 55 (14 - 165) | 0.6 |
| hsTnT (ng/L)* | 18 (4 - 132) | 8 (4 - 64) | <0.001 | 17 (4 - 132) | 6 (4 - 61) | <0.001 |
| NT-proBNP (ng/L)^ | 157.4 (8.6 - 9782.0) | 47.7 (5.0 - 1628.0) | <0.001 | 175.7 (5.8 - 9782.0) | 36.9 (5.0 - 1628.0) | <0.001 |
| uACR (mg/mmol)^ | 5.2 (0.4 - 484.7) | 1.3 (0.2 - 373.7) | <0.001 | 5.3 (0.3 - 484.7) | 0.95 (0.2 - 129.1) | <0.001 |
| WCC (10*9/L)* | 7.7 (2.8) | 7.4 (2.1) | 0.602 | 8.0 (2.4) | 7.1 (2.1) | 0.008 |
| Haemoglobin (g/dL)* | 12.1 (1.8) | 13.7 (1.5) | <0.001 | 12.8 (1.7) | 13.8 (1.4) | <0.001 |
| Platelet Count (10*9/L)* | 262 (96) | 246 (73) | 0.0305 | 248 (86) | 249 (72) | 0.898 |
| Rate of Change in Renal Function | | | | | | |
| Absolute Change (mL/min/1.73m^2^/year)* | -4.959 (1.770) | -1.128 (1.581) | <0.001 | -3.504 (1.739) | -0.598 (1.531) | <0.001 |
| Percentage Change (% change/year)* | -9.817 (5.145) | -1.956 (3.230) | <0.001 | -7.270 (4.217) | -0.573 (2.593) | <0.001 |

**Supplementary Table 8:** Comparison of baseline biochemical and haematological parameters in decliners versus non-decliners classified based on absolute decline (≤-3.5mL/min/1.73m^2^/year) or percentage decline (≤-3.3%/year) in renal function.

Dp-ucMGP: dephosphorylated-uncarboxylated Matrix Gla-Protein; HbA_1c_: glycated haemoglobin; CRP: C-reactive protein; eGFR: estimated glomerular filtration rate (CKD-EPI); adj. calcium: adjusted calcium, ALP: alkaline phosphatase; ALT: alanine aminotransferase; GGT: gamma-glutamyl transferase; HDL-C: high-density lipoprotein cholesterol; LDL-C: low-density lipoprotein cholesterol; T4: thyroxine; TSH: thyroid stimulating hormone; iPTH: intact parathyroid hormone; 25 (OH) D: 25-hydroxycholecalciferol; hsTnT: high-sensitivity troponin T; NT-proBNP: n-terminal pro b-type natriuretic peptide; WCC: white cell count; uACR: urine albumin:creatinine ratio. ^ Median (minimum to maximum); * mean (standard deviation). ^¥^ p-values represent significance levels for comparisons between decline and no-decline groups – students t-test for parametric and Mann Whitney test for non-parametric data.

**Supplementary Result:**

The manufacturer’s claims for time to first test result and throughput were verified. From successful calibration and assay initiation, the first result was released within 64 minutes and test throughput was confirmed at 80 reportable tests per hour. The minimum plasma volume required to perform the assay was 150μL.

**References:**

1. Tremblay AJ, Morrissette H, Gagne JM, Bergeron J, Gagne C, Couture P. Validation of the Friedewald formula for the determination of low-density lipoprotein cholesterol compared with beta-quantification in a large population*.* Clin Biochem 2004;37:785-90.

2. Clinical Laboratory Standards Institute. User Verification of Precision and Estimation of Bias. EP15 A3. 3rd edition, 2014

3. CLSI. Clinical and Laboratory Standards Institute (CLSI). Defining, Establishing, and Verifying Reference Intervals in the Clinical Laboratory; Approved Guideline—Third Edition CLSI document C28-A3 (ISBN 1-56238-682-4)*.* 2008.
